# Resveratrol and dexamethasone have cell-specific effects on the circadian clock but not on the rhythm of mitochondrial function in the fetal heart

**DOI:** 10.1007/s13105-026-01152-8

**Published:** 2026-02-16

**Authors:** Dmytro Semenovykh, Kateryna Semenovykh, Martin Sládek, Alena Sumová

**Affiliations:** 1 Laboratory of Biological Rhythms, Institute of Physiology of the Czech Academy of Sciences, Videnska 1083, 14220 Prague, Czech Republic; 2 Department of Physiology, Faculty of Science, Charles University, Vinicna 7, 12844 Prague, Czech Republic

**Keywords:** Fetal cardiomyocytes, Fetal cardiac fibroblasts, Resveratrol, Dexamethasone, Circadian rhythm, Mitochondria

## Abstract

Proper development of the heart during the fetal period is a prerequisite for health in later life. It was shown that maternal supplementation with stilbenoid resveratrol (RES) is beneficial for cardiovascular function. Synthetic glucocorticoid dexamethasone (DEX) is commonly used in the prenatal treatment of respiratory distress syndrome. RES affects the circadian clock in various tissues but its effect on the fetal heart has not been studied. We hypothetized that RES may affect the the circadian clock via modulation of the glucocorticoid signaling and/or mitochondrial function. Therefore, we tested the effects of different concentrations of RES and synthetic glucocorticoid dexamethasone (DEX) on the circadian clock, energy metabolism and mitochondrial function in fetal cardiomyocytes (CMCs) and cardiac fibroblasts (FBs). We found that RES affects the clock in both CMCs and FBs by increasing the stability of the clock protein PER2. In CMCs, the effect was mediated via the adenylyl cyclase signaling pathway. RES modulated DEX-induced effects on the circadian clock in CMCs and FBs. We were able to detect a circadian rhythm in mitochondrial function in fetal heart cells, which was confirmed by ATP and resazurin assays as well as visualization of the mitochondrial network and reactive oxygen species (ROS). Interestingly, we found that both drugs shifted the phase of fetal heart clock, but had no effect on the phase of mitochondrial rhythmicity, indicating a possible uncoupling of circadian and mitochondrial rhythms in fetal CMCs and FBs. Overall, our data revealed fetal heart-specific effects of RES on the circadian clock through the stabilization of PER2 protein and its ability to modulate DEX-induced effects on the clock.

**Graphical Abstract:**

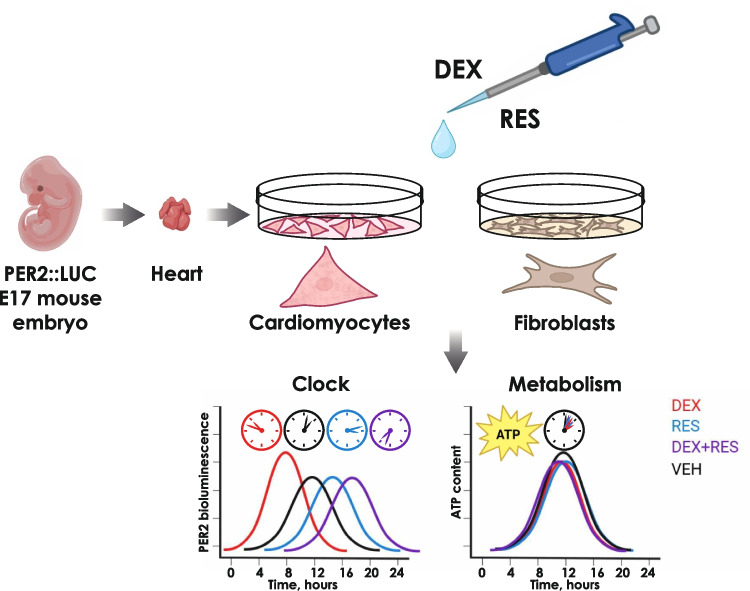

**Supplementary Information:**

The online version contains supplementary material available at 10.1007/s13105-026-01152-8.

## Introduction

The fetal cardiovascular system plays a critical role in distributing highly oxygenated blood from the placenta to all organs and tissues. During the prenatal period, the heart develops intensively through the proliferation of both cardiac myocytes and non-myocyte cells [51]. Close interaction between mother and embryo is essential for normal development and a successful transition to the postnatal period.

One of the important factors during the prenatal phase is the exposure and adaptation of the future offspring to the circadian rhythmicity of the maternal organism. The circadian rhythmicity of the mother is driven by the circadian clock in the suprachiasmatic nuclei of the hypothalamus (SCN) (reviewed in [30]), which generates rhythmic signals with a period of approximately 24 hours to control many processes in the body. This provides the organism with adaptation to daily changes in the environment, such as light, food availability and temperature [53]. The SCN clock sends rhythmic signals to the clocks in various peripheral tissues that rhythmically drive local cellular processes [17]. These peripheral clocks include all cell types within the cardiovascular system [6, 20, 21, 47, 75].

At the cellular level, the circadian rhythm is driven by transcriptional-translational feedback loops (TTFLs) (reviewed in [74]). At the core of the mechanism, aryl hydrocarbon receptor nuclear translocator-like protein 1 (ARNTL; also known as BMAL1) and circadian locomotor output cycles protein kaput (CLOCK) proteins form a complex that activates the transcription of *Cryptochrome* 1 and 2 (*Cry1* and *Cry2*) and *Period* 1, 2 and 3 (*Per1*, *Per2* and *Per3*, respectively) [19]. CRY and PER accumulate in the cytoplasm and form heterodimers that inhibit BMAL1-CLOCK transcriptional activity and create negative feedback [66]. Additional feedback loops are employed to maintain the accuracy and robustness of the circadian signal (reviewed in [42]).

In the mouse hearts, clock genes are not expressed rhythmically during the early embryonic stages (E10-E12), but begin to gain circadian expression patterns by the later stages of fetal development (E17-E19) [78]. Circadian rhythms play a crucial role in regulating fetal heart metabolism and function [27, 39]. During the prenatal stage, mothers are able to communicate with the developing fetal clocks via multiple pathways [28], including neurohumoral (reviewed in [71]) and metabolic [67] signals, however, their mutual interaction has been poorly studied.

For the humoral pathways, glucocorticoid signaling was suggested to play such a role, as dexamethasone (DEX) significantly affected the circadian clock and facilitated the development of rhythmicity in the fetal SCN in vitro [9]. The effect of glucocorticoid signaling on the circadian clock in the fetal heart has not been studied, despite the fact that prenatal application of DEX has been used to reduce the respiratory distress syndrome in preterm neonates in clinics (reviewed in [82]). Changes in metabolic state represent other pathways potentially involved in the communication between the mother and the fetal clocks. Indeed, the feeding regime during pregnancy can provide a significant signal to the circadian clock in the SCN of the fetuses [28, 55, 67]. At the molecular level, there is a growing evidence that the circadian rhythm is linked to metabolism via epigenetic changes such as protein deacetylation by the enzyme Sirtuin 1 (SIRT1) [7]. One of the key circadian regulators, CLOCK, has intrinsic acetyltransferase activity that enables the acetylation of histone [18] and non-histone targets [31]. The NAD+-dependent deacetylase SIRT1 acts as a counterbalance to CLOCK and participates in the regulation of the circadian rhythm by deacetylating the PER2 protein [2]. Thus, epigenetic regulators, such as SIRT1, are plausible mediators of maternal metabolic signals to the fetal heart.

Mitochondrial content is well known to be highly dependent on tissue type, with mtDNA, mRNA, enzymatic activity, respiration rates and protein content varying considerably [23, 25, 36]. Furthermore, parts of the mitochondrial proteome of heart, liver and skeletal muscle are organ-specific [25]. Importantly, many mitochondrial proteins exhibit a circadian rhythm in their expression [52, 62], and circadian rhythmicity is also typical for some genes involved in mitochondrial function in the murine heart [41]. The subsarcolemmal but not intramyofibrillar fraction of mitochondria was decreased in the heart of *Clock*^*∆19*^-deficient mice [8]. In particular, it was demonstrated that the circadian-controlled mitochondrial fusion-fission [65] is impaired in diabetic cardiomyopathy [76]. Interestingly, glucocorticoid and metabolic pathways may interact because prenatal treatment with DEX increased mitochondrial catalase activity in the sheep heart [81].

All these findings highlight the need to investigate the molecular mechanisms regulating the circadian clock in the fetal heart. To address this, we used resveratrol (RES), a natural polyphenol that acts as an enhancer of SIRT1 activity [34], to study the involvement of epigenetic pathways in modulating the circadian clock in the fetal heart. Because glucocorticoids are significant maternal signals to the fetus, we used dexamethasone (DEX) to examine the interaction between glucocorticoid and metabolic pathways affecting the fetal heart, specifically regarding clock maturation and mitochondrial function.Our first aim was to determine whether exposure to glucocorticoids alters the development and circadian regulation of core clock components and mitochondrial dynamics selectively in two different cell types, fetal mouse cardiomyocytes (CMCs) and cardiac fibroblasts (FBs). The effect of RES in fetal mouse CMCs and FBs has not been investigated; therefore, we hypothesized that RES can modulate DEX-induced effects on circadian and mitochondrial rhythms differently in these two fetal heart cell types. The second aim was to determine whether RES can modulate the effects of DEX on circadian and mitochondrial rhythms via SIRT1 and whether glucocorticoid and epigenetic perturbations may affect mutual coupling of circadian and mitochondrial processes..

Our findings support the effect of maternal glucocorticoid signals on the circadian rhythm in the fetal heart and identify the SIRT-dependent pathway as a potential modulator of glucocorticoid-induced alterations in clock-mitochondria coupling.

## Materials and Methods

### Experimental animals

Adult male and female *mPer2*^*Luc*^ mice (strain B6.129S6-Per2tm1Jt/J, JAX, USA; a colony maintained at the Institute of Physiology of the Czech Academy of Sciences) were housed individually under a 12 h light and 12 h dark (LD12:12) cycle (lights on between 0700 h and 1900 h) in a temperature-controlled facility at 23 °C ± 2 °C, with food and water available *ad libitum*. Vaginal smears from female mice were examined to identify the phase of the estrous cycle. Mating occurred on the night of proestrus, and the following morning, when the vaginal smears were sperm-positive, was designated as day 0 of embryonic development (E0). Pregnant *mPer2*^*Luc*^ mice were sacrificed by cervical dislocation at gestational age E17. To record the PER2-driven bioluminescence signal and construct the phase response curves, seven fetuses of the same litter of six pregnant mice for each method were sacrificed to collect the hearts. For the RT-qPCR assay, at least seven fetuses from each litter of four pregnant mice were sacrificed to collect the hearts. For the study of the rhythm of mitochondrial function and PER2 decay, we used at least seven embryos from each of three pregnant mice per experimental method. Male and female hearts from a single litter were pooled for the isolation of CMCs and FBs. After the collection of the hearts, they were placed in a cold PBS buffer and further processed for the isolation of embryonic cardiomyocytes and fibroblasts.

Experiments are performed in accordance with a valid experimental project reference number AVCR 8271/2022 SOV II. The project is approved by the Animal Care and Use Committee of the Institute of Physiology of the Czech Academy of Sciences, as well as by the Resort Professional Commission of the CAS for Approval of Projects of Experiments on Animals. The animals are housed in facilities accredited by the Czech Ministry of Agriculture. Experiments are carried out under veterinary supervision, complying with Act No. 246/1992 Coll. and Decree No. 419/2012 Coll., implementing Directive 2010/63/EU of the European Parliament and of the Council regarding the protection of animals used for scientific purposes. The 3Rs principles are applied to the maximum extent possible.

### Cell isolation and treatments

On embryonic day 17 (E17), CMCs and FBs were isolated by enzymatic digestion as previously described [60]. Briefly, after pregnant mice were sacrificed by cervical dislocation, embryos were placed into ice-cold phosphate-buffered saline. Fetal hearts were excised and transferred to an isolation buffer containing 100 mM NaCl, 10 mM KCl, 1.2 mM KH2PO4, 4 mM MgSO4, 50 mM taurine, 20 mM glucose, and 10 mM HEPES (pH 6.9). Ventricles were dissected and placed into an isolation buffer containing 2 mg/ml pancreatin (Sigma-Aldrich, St. Louis, MO, USA) and 2 mg/ml collagenase type II (Sigma-Aldrich, St. Louis, MO, USA). The tissue was digested for 50 min at 37°C. After mechanical dissociation, cell suspension was centrifuged at 750 g for 5 min with further resuspension in high-glucose DMEM (Thermo Fisher Scientific, Waltham, MA, USA) supplemented with 10% fetal bovine serum (Sigma-Aldrich, St. Louis, MO, USA), 1% GlutaMAX (Thermo Fisher Scientific, Waltham, MA, USA), 1% MEM non-essential amino acids (M7145, Sigma-Aldrich, St. Louis, MO, USA) and 1% penicillin/streptomycin solution (P4333, Sigma-Aldrich, St. Louis, MO, USA). The obtained cells were pre-plated for 2 h in T25 cell culture flasks at 37 °C in a humidified 5% CO2 incubator in order to separate CMCs and FBs. Purity of isolated cells was determined using sarcomeric alpha actin antibody (marker of cardiomyocytes) using the immunofluorescence method (A7811, Sigma-Aldrich, St. Louis, MO, USA). CMCs were counted and plated on gelatin-covered plates. After reaching confluence, FBs were trypsinized using Trypsin/EDTA Solution (Sigma-Aldrich, St. Louis, MO, USA) and seeded on plates. Both CMCs and FBs were counted with a hemocytometer and trypan blue. Next, cells were seeded at a density of 60,000 cells/100 μl.

### Experimental design

Before the treatments, cells were synchronized by serum shock (50% horse serum/DMEM for 1.5 h at 37 °C) in all experiments [6, 20, 21, 87]. Thereafter, the medium was changed to the air-buffered recording medium [85] supplemented with 10% fetal bovine serum (Sigma-Aldrich, St. Louis, MO, USA), 1% GlutaMax (Thermo Fisher Scientific, Waltham, MA, USA), 1% penicillin/streptomycin (Sigma-Aldrich, St. Louis, MO, USA) and 1% MEM non-essential amino acids. A concentration of 0.1 mM D-Luciferin (Biosynth, Staad, Switzerland) was added to an air-buffered recording medium prior to bioluminescence recording. CMCs and FBs were treated with 100, 10 or 1 μM DEX (D4902, Sigma-Aldrich, St. Louis, MO, USA) or with media alone in the presence or absence of 5 or 25 μM RES ((R5010, Sigma-Aldrich, St. Louis, MO, USA). Both DEX and RES were dissolved in DMSO. Overall, there were four treatment groups: 1) DMSO-treated vehicle (VEH) with a final concentration of 0.1% DMSO in the medium; 2) DEX-treated; 3) RES-treated; and 4) DEX and RES co-treated groups.

### Resazurin assay

To estimate innate metabolic activity (NADH level), we performed the resazurin assay according to the manufacturer’s instructions. Resazurin (R7017, Sigma-Aldrich, St. Louis, MO, USA) was added to a concentration of 0.01 mg/ml to the air-buffered recording medium with treatment upon reaching 0, 6, 12, 18 and 24 hours time points. After 3 hours, the medium was collected for absorbance measurement at 570 nm and 600 nm using the Cytation 3 Cell Imaging Multi-Mode Reader (Agilent Technologies, Santa Clara, CA, USA). For each well, the absorbance percentage reduction was calculated using the following equation: Resazurin reduction percentage = [(εOX600nm × A570nm_t3 − εOX570nm × A600nm_t3)/(εRED570nm × A600nm_NC − εRED600nm × A570nm_NC)] ×100. Here, εOX denotes the molar extinction coefficient of oxidized Resazurin at 570 nm/600 nm, εRED is the molar extinction coefficient of reduced Resazurin at 570 nm/600 nm, and A denotes the measured absorbance at 570 nm/600 nm of negative control (NC) and after 3 h of incubation (t3).

### ATP assay

The assay was performed utilizing the CellTiter-Glo Luminescent Cell Viability Assay kit (G7571, Promega, Madison, WI, USA), according to the manufacturer’s instructions. Cells were seeded at 60,000 cells/100 μl in the 96-well white plate (SPL Life Sciences, Pocheon-si, Gyeonggi-do, Korea) and incubated at 37 ^o^C, 5% CO2 for 24 h before treatment. After the start of the treatment, separate plates were processed every 6 hours within a 24-hour period. The wells of each plate were washed with phosphate-buffered saline (PBS), and, subsequently, CellTiter-Glo reagents were mixed at a 1:1 ratio with PBS. The solution was incubated for 10 min at room temperature, followed by luminescence measurements using the Cytation 3 Cell Imaging Multi-Mode Reader (Agilent Technologies, Santa Clara, CA, USA).

### Bioluminescence recordings

Cells were synchronized by serum shock for 1.5 hours. Immediately after that, serum shock was replaced by the air-buffered recording medium with the addition of 0.1 mM D-Luciferin (FL08608, Biosynth, Switzerland). Bioluminescence recordings in CMCs and FBs cell cultures were conducted in the Luminoskan Ascent plate reader (Thermo Fisher Scientific, Waltham, MA, USA) with frequency of readings every 15 minutes at 37 ^o^C. Recorded traces were analyzed using an adapted Python script “per2py” (https://github.com/johnabel/per2py) for automated analysis of circadian bioluminescence data to determine period, amplitude, decay and trend parameters [46]. The parameter period reflects the duration of one complete circadian cycle. Amplitude is the difference between the highest point of circadian oscillation and mesor. The decay parameter is the dynamics of the bioluminescence damping. The trend reflects the mean PER2::LUC level throughout the recorded bioluminescence.

In order to construct a phase response curve (PRC), cells were seeded on 35-mm petri dishes. After completion of synchronization, 1 ml of the air-buffered recording medium supplemented with 0.1 mM D-Luciferin was added to CMCs and FBs, with the subsequent recording of bioluminescence traces using a Lumicycle (Actimetrics, Wilmette, IL, USA) with frequency of readings every 10 minutes at 37 ^o^C. Bioluminescence was monitored for 2 days. On the third day, cells were treated every hour within a 24-hour period. Both CMCs and FBs were treated with 100 μM DEX, 25 μM RES, a 100 μM DEX and 25 μM RES co-treatment or VEH, followed by the subsequent recording of bioluminescence traces for 2 more days in white hermetically sealed plates. The data were processed with LumiCycle Analysis software (Actimetrics, Wilmette, IL, USA) and adjusted by subtracting the baseline with a 24-hour running average and fitted to a damped sine wave to determine the period. The phase shift was quantified by fitting it to two full circadian cycles and designated as a phase advance (+) or a delay (-). The PRCs were built by plotting the phase shift as a function of treatment time.

### PER2 degradation assay

After synchronization and treatment with DEX, RES or DEX+RES, cells were placed in the Luminoskan Ascent apparatus with subsequent recording of bioluminescence traces. For this experiment, cells were treated with 100 μM DEX, RES (5, 25, 50 and 75 μM), DEX and RES co-treatment and VEH. Additionally, we used 150 μM SIRT6 inhibitor OSS_128167 (Cat#HY-107454, MedChemExpress, Monmouth Junction, NJ, USA) and 10 μM of the adenylyl cyclase inhibitor (MDL-12,330A hydrochloride, Sigma-Aldrich, St. Louis, MO, USA), which were added after synchronization to cell cultures to elucidate the effect of RES on PER2 stability. After the beginning of the treatment, cells were placed in the Luminoskan Ascent apparatus to record bioluminescence traces in all treatment groups. Next, FBs and CMCs were treated with an inhibitor of translation - 40 μg/ml cycloheximide (CHX) (Sigma-Aldrich, St. Louis, MO, USA) upon reaching the first peak of PER2-driven bioluminescence. The bioluminescence recording continued next 10 h. Afterwards, cells were washed with PBS, placed into fresh recording medium, and immediately recorded in the Luminoskan Ascent apparatus for 12–24 h. Degradation curve data obtained after the first 9 h of recording were fitted to one-phase exponential decay curves to obtain the half-life and K value.

### Total RNA isolation and RT-qPCR

CMCs and FBs were seeded (60,000 cells/100 μl) with subsequent synchronization using serum shock as previously described. Medium with 100 μM DEX, 25 μM RES, 100 μM DEX+25 μM RES or VEH treatment was added right after the end of the synchronization. Then, after 24 h of treatment, cells were washed with PBS and harvested in RLT lysis buffer. Subsequently, total RNA was isolated using the RNeasy Micro kit (74104, Qiagen, Hilden, Germany) according to the manufacturer’s instructions. The concentration and quality (260/230 ratio) of RNA samples were measured using NanoDrop™ One Microvolume UV-Vis Spectrophotometer (Thermo Fisher Scientific, Waltham, MA, USA). Total RNA (250 ng for CMCs and 500 ng for FBs) was transcribed into cDNA utilizing the High-Capacity cDNA Reverse Transcription Kit (4368814, Thermo Fisher Scientific, Waltham, MA, USA). We used the following temperature cycle: reverse transcription initiation (25°C) 10 minutes, reverse transcription reaction (37°C) 120 minutes, enzyme inactivation (85°C) 5 minutes, and hold (4°C). Subsequently, the obtained cDNA was diluted 1:10 and 2 μl were used for further RT-qPCR reaction (7 μl total volume). The obtained cDNA samples were quantified by the LightCycler 480 Real-Time PCR System (Roche, Basel, Switzerland) using PowerUp SYBR Green Master Mix (A25741, Thermo Fisher Scientific, Waltham, MA, USA) using the following temperature cycles: denaturation (95°C) 5 s, annealing and amplification (60°C) 30 s, 45x. The LightCycler 480 software was used for the calculation of the Ct of each reaction. Quantification was performed using the ΔΔCt method in Microsoft Excel. Relative expression was calculated by normalizing the expression to the housekeeping genes for CMCs (*Gapdh*, *Hprt*, *Pgk1*) and FBs (*Hprt*). A list of primers is provided in the Supplementary Table 1.

### Immunofluorescence

The cells were seeded on glass coverslips with treatment starting the next day. After the beginning of treatment, cells were collected every 6 h with a total of five time points (0, 6, 12, 18 and 24 h after the beginning of treatment). After reaching a certain time point, cells were incubated with 100 nM Mitotracker Red with 579/599 nm excitation/emission wavelength range, 5 μM CellROX Green Reagent with 485/520 nm excitation/emission wavelength range (Thermo Fisher Scientific, Waltham, MA, USA), and DAPI with 350/465 nm excitation/emission wavelength range (H-1200-10, Vector Laboratories, Newark, CA, USA) for 30 min at 37 °C in a humidified 5% CO2 incubator. Then, cells were washed twice with PBS and fixed with 4% paraformaldehyde for 10 min at room temperature. After that, coverslips were mounted on glass slides with fluorescent mounting medium. Confocal images were recorded using a Leica SP8 single-photon confocal microscope. The acquired mitochondrial networks were analyzed using the Mitochondria Analyzer ImageJ plugin.

### Statistical Analysis

Statistical analyses were conducted using GraphPad Prism 8 (GraphPad Software, Boston, MA, USA). The RT qPCR data and bioluminescence period, amplitude, decay and trend parameters were compared by 1-way ANOVA with Tukey’s multiple comparison test. Cosinor analysis was used for investigation of ATP, resazurin, reactive oxygen species (ROS), mitochondrial dynamics and mitochondrial membrane potential (MMP) rhythms [15]. The validation of sinusoidal rhythmicity was done using the goodness-of-fit parameter (Supplementary Table 2). Binned PRC and acrophase data were analyzed by 1-way ANOVA with Dunnett's multiple comparisons. The acrophase of PER2 bioluminescence was examined for comparison with the acrophase of the ATP rhythm. PER2 half-life values were compared between all experimental groups using 1-way ANOVA with Tukey’s multiple comparisons test. First, we calculated the relevant parameters of the circadian rhythm for ATP, resazurin, mitochondrial network, ROS, and MMP assays using cosinor analysis. Subsequently, we applied 2-way ANOVA (for ATP and resazurin assays) and 2-way mixed ANOVA analysis (for mitochondrial network) with Greenhouse-Geisser correction, followed by Tukey’s post-hoc test, to compare the parameters across experimental groups. For statistical analysis of ROS and MMP assays, 2-way mixed ANOVA analysis with Sidak’s multiple comparison test was used. Data are expressed as the mean ± SD. A *P-value* of < 0.05 was considered significant. All experiments were performed with at least three biological replicates.

## Results

### RES 25 μM but not 5 μM prevents the effect of DEX on the rhythm in PER2::LUC bioluminescence in CMCs and FBs

CMCs and FBs isolated from hearts of *mPer2*^*Luc*^ mouse fetuses at E17 were treated with one of two concentrations of RES (5 and 25 μM) and DEX (100, 10, and 1 μM) (Supplementary Fig. 1). A bioluminescence plate reader recorded their circadian rhythms for 3 days. We found that the effect of RES, DEX, and DEX + RES co-treatment was cell-type-dependent (for statistics, see Supplementary Table 2).

**Fig. 1:**
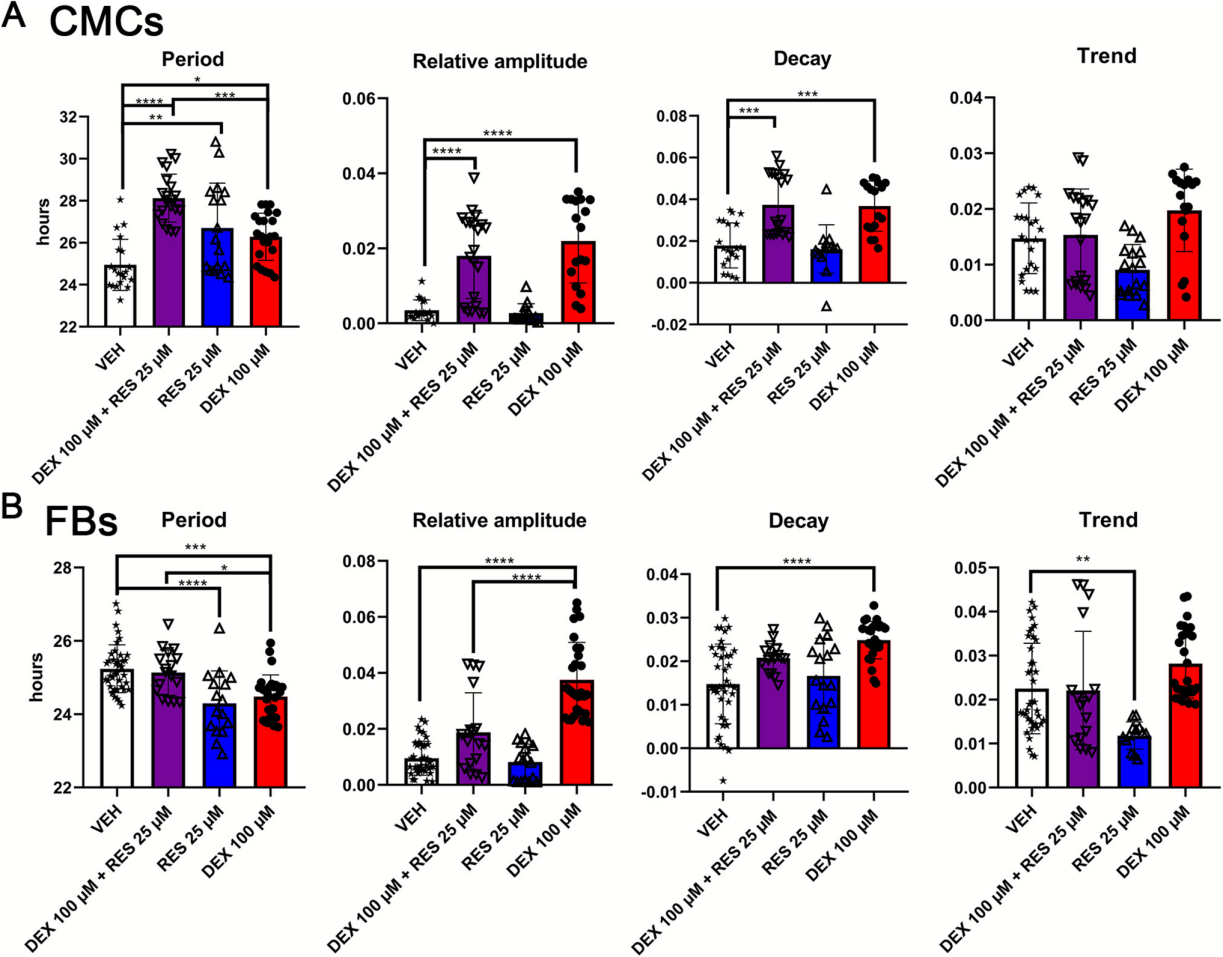
Co-treatment with 25 μM RES abolishes DEX-induced changes in oscillatory parameters in FBs but not CMCs. Isolated cells were seeded at 60,000 cells/100 μl and, after synchronization, were treated with 100 μM DEX, co-treated with 25 μM RES, and VEH. The bioluminescence parameters alterations were evaluated by comparing the period, amplitude, decay, and trend of the experimental groups in CMCs **(A)** and FBs **(B)**. Cells were isolated from pooled seven or more fetal hearts from each of six pregnant mice. Data were compared using 1-way ANOVA with Tukey’s multiple comparison test and presented as individual ratios and mean ± SD. Asterisks show the results of Tukey's multiple comparisons test between treatment groups. ∗*P* < 0.05, ∗∗*P* < 0.005, ∗∗∗*P* < 0.0005, ∗∗∗∗*P* < 0.0001

Treatment with 5 μM RES did not result in any significant changes in the period in comparison to VEH in both CMCs and FBs (P > 0.999 and *P = 0.936,* Supplementary Fig. 1). However, 25 μM RES had a significant effect on the period in both cell types. In CMCs, it prolonged the period (*P* = 0.0019, Fig. 1A), whereas in FBs, it shortened the period (*P* < 0.0001, Fig. 1B). In turn, 100 μM DEX prolonged the period in CMCs (*P* = 0.0148; Fig. 1A). In contrast, the period in FBs was shortened after 100 μM DEX (*P* < 0.0001, Fig. 1B), 10 μM DEX and 1 μM DEX (*P* = 0.0006 and *P* = 0.0005, Supplementary Fig. 1B). Despite the opposite effect of 25 μM RES on the period of both cell types, its co-treatment with 100 μM DEX resulted in a prolonged period in comparison with DEX treatment alone in both CMCs (*P* = 0.0004, Fig. 1A) and FBs (*P* = 0.0011, Fig. 1B).

The relative amplitude was not affected by 5 μM RES (P > 0.999*,* Supplementary Fig. 1) and 25 μM RES (*P* = 0.994*,* Fig. 1). In both CMCs and FBs, all used concentrations of DEX increased the relative amplitude of bioluminescence. The co-treatment of DEX (100, 10, and 1 μM) and 5 μM RES had a similar effect on amplitude as DEX treatment alone in CMCs (P > 0.999; Supplementary Fig. 1). Similarly, co-treatment with 5 μM RES did not lead to any changes in the DEX-induced amplitude in FBs (P > 0.999; Supplementary Fig. 1). However, co-treatment of 25 μM RES with DEX (1 μM, 10 μM and 100 μM) resulted in a significant decrease in amplitude and thus prevented the DEX-induced rise of the amplitude. The results show a dose-dependent and cell-type-specific effect of RES, as there were no changes in amplitude with co-treatment of DEX (100, 10, and 1 μM) and 25 μM RES in CMCs (P > 0.999, Supplementary Fig. 1).

The decay parameter was affected by 5 μM RES in a cell-type-dependent manner. In CMC, decay was not changed after 5 μM RES (P > 0.999, Supplementary Fig. 1A) and 25 μM RES (*P* = 0.977, Fig. 1A) treatment. In FBs, 5 μM RES decreased the decay (*P =* 0.0002, Supplementary Fig. 1B). However, treatment of FBs with the higher dose of RES (25 μM) had no effect (*P = 0.8106,* Fig. 1B). The decay was altered after DEX and RES treatments. DEX application led to an increase in decay in both CMCs (Fig. 1A) and FBs (Supplementary Table 2; Fig. 1B). In CMCs, co-treatment with 5 and 25 μM RES did not result in any changes in DEX-induced decay. However, 25 μM RES co-application with 1 μM DEX led to a slight increase in decay comparable to DEX treatment alone (*P* = 0.0457; Supplementary Fig. 1A). There were no statistically significant differences between the DEX groups and the DEX + RES co-treatment groups in FBs (Supplementary Fig. 1B).

Whereas 5 μM RES did not alter the trend (*P* = 0.999; Supplementary Fig. 1B), treatment with 25 μM RES decreased the trend in FBs (*P =* 0.0046). In CMCs, DEX + RES co-treatment did not induce any changes in the trend. In FBs, the trend in the DEX + RES co-treated group was significantly decreased in comparison to the VEH and the DEX group (Supplementary Table 2, Fig. 1B). Interestingly, while the trend was decreased by 25 μM RES in both CMCs and FBs, it was reduced only in FBs after co-treatment with DEX.

Altogether, our results showed that 5 μM RES significantly decreased the dynamics of the decline in the amplitude of bioluminescence rhythms (decay) only in FBs. However, 25 μM RES changed the period (with opposite effect) and trend in CMCs and FBs. Our data show that DEX alone induced cell type-dependent changes in period. Co-treatment of DEX with 25 μM RES prevented DEX-induced changes in the period and amplitude of the circadian rhythm in FBs.

### RES prolongs the PER2::LUC half-life via the cAMP-dependent pathway in the fetal CMCs

Since DEX and RES induced significant changes in the period of PER2 bioluminescence, we decided to test the effects of both treatments on PER2 protein stability using a degradation experiment. After the PER2::LUC bioluminescence signal in the CMC and FB cultures reached the peak, we blocked de novo translation with the translation inhibitor cycloheximide (40 μg/ml) and recorded the dynamics of bioluminescence decline as a proxy of PER2 degradation.

**Fig. 2:**
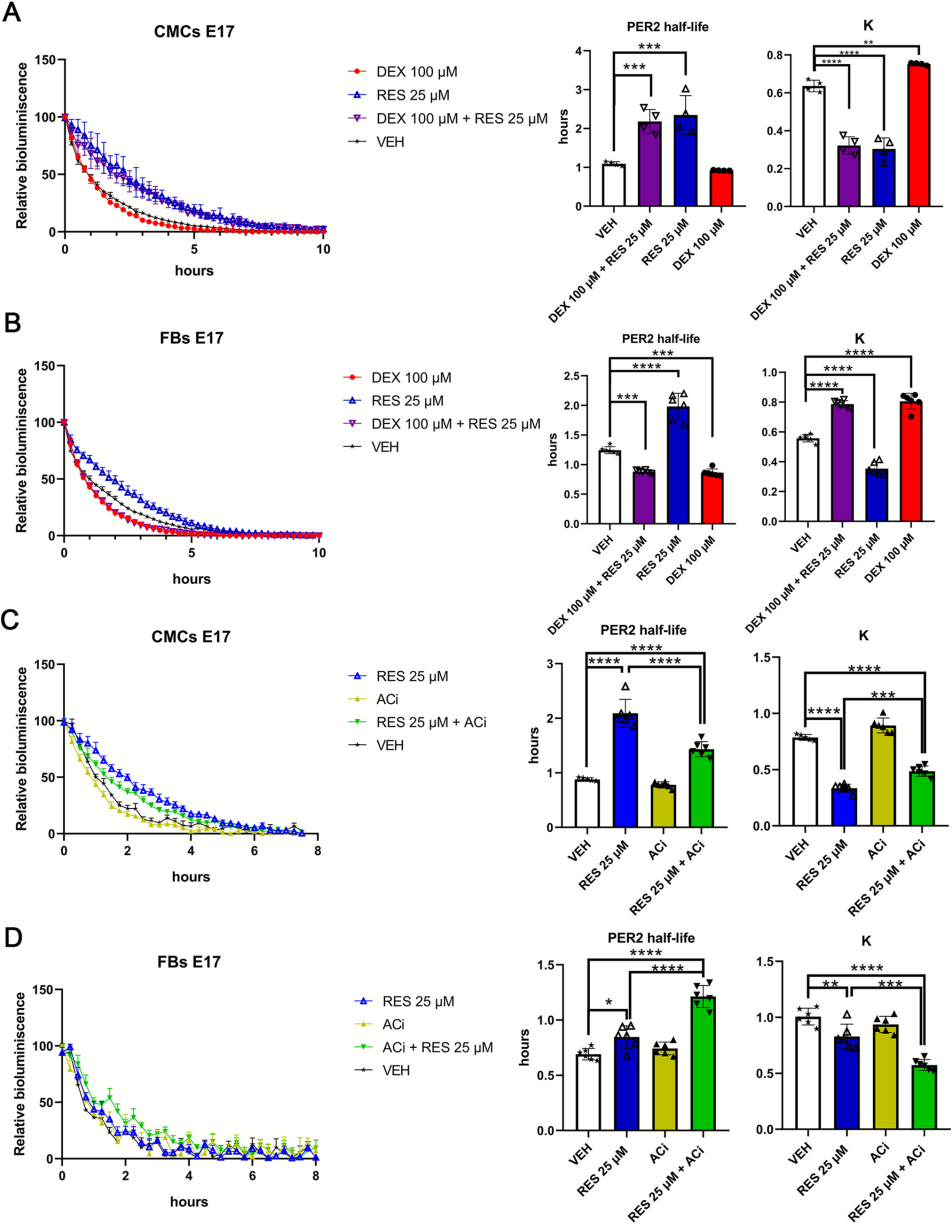
RES treatment increased PER2 stability in E17 CMCs and FBs. Cells were isolated from a pooled seven or more E17 hearts from each of three pregnant mice and seeded at 60,000 cells/100 μl. CMCs and FBs were treated with DEX, RES, co-treatment or VEH (**A** and **B**). To reveal the PER2 stabilizing effect, CMCs and FBs were treated with RES, ACi co-treatment and VEH in further experiments (**C** and **D**). Upon reaching a peak of the bioluminescence, cells were treated with cycloheximide (CHX, 40 μg/ml) that caused the termination of the translation with the subsequent recording of PER2 degradation curve in both CMCs and FBs (**A**, **B**, **C** and **D** left graphs). PER2 degradation was quantified by fitting exponential decay curves to the first 10 h of luminescence data post-CHX treatment. A half-life and K parameter between treatment groups was compared using 1-way ANOVA with Tukey’s multiple comparisons test (**A**, **B**, **C** and **D** right graphs). ∗*P* < 0.05, ∗∗*P* < 0.005, ∗∗∗*P* < 0.0005, ∗∗∗∗*P* < 0.0001

In CMCs, DEX treatment did not result in significant changes in PER2::LUC half-life compared to the VEH (Supplementary Table 2; *P* = 0.227; Fig. 2A). Interestingly, 25 μM RES prolonged the half-life of PER2::LUC (*P* = 0.0003). CMCs co-treated with both 100 μM DEX and 25 μM RES showed increased PER2::LUC half-life compared to DEX treatment alone (*P* = 0.0003).

In FBs, DEX led to a decrease in PER2::LUC half-life compared to the VEH group (Supplementary Table 2; *P* = 0.0001; Fig. 2B). Similarly to CMCs, 25 μM RES had a strong PER2::LUC stabilizing effect in comparison to VEH (*P* < 0.0001). Moreover, using a range (5, 25 and 50 μM) of RES concentrations, we showed a dose-dependent effect on PER2 half-life in both CMCs and FBs (Supplementary Fig. 2). No statistically significant difference was found between DEX alone and the co-treatment with 25 μM RES (*P* = 0.993). Overall, we found a dose-dependent effect of RES on PER2 stability.

We aimed to elucidate the mechanism by which RES prolongs PER2 half-life. We first tested the effect of RES on SIRT6, because it was previously reported that RES leads to increased SIRT6 expression in HeLa cells [58]. However, co-treatment with selective SIRT6 inhibitor OSS_128167 (Cat#HY-107454, MedChemExpress, USA) did not reduce the increased stability of PER2 resulting from RES (Supplementary Fig. 3). Next, we tested the involvement of the cAMP signaling pathway by co-treatment of RES with an adenylyl cyclase inhibitor (ACi, MDL-12,330A hydrochloride). In CMCs, co-incubation of RES and ACi led to the partial decrease of RES-increased PER2 half-life (Supplementary Table 2; *P* < 0.0001; Fig. 2C). In FBs, the effect of ACi varied from an increase of half-life to the absence of any effect on PER2 stability (*P* < 0.0001; Fig. 2D). Thus, RES increased PER2 stability, at least partially, via the adenylyl cyclase pathway in CMCs but not in FBs.

### RES and DEX shift the phase of circadian clocks but not ATP rhythm in CMCs and FBs

**Fig. 3:**
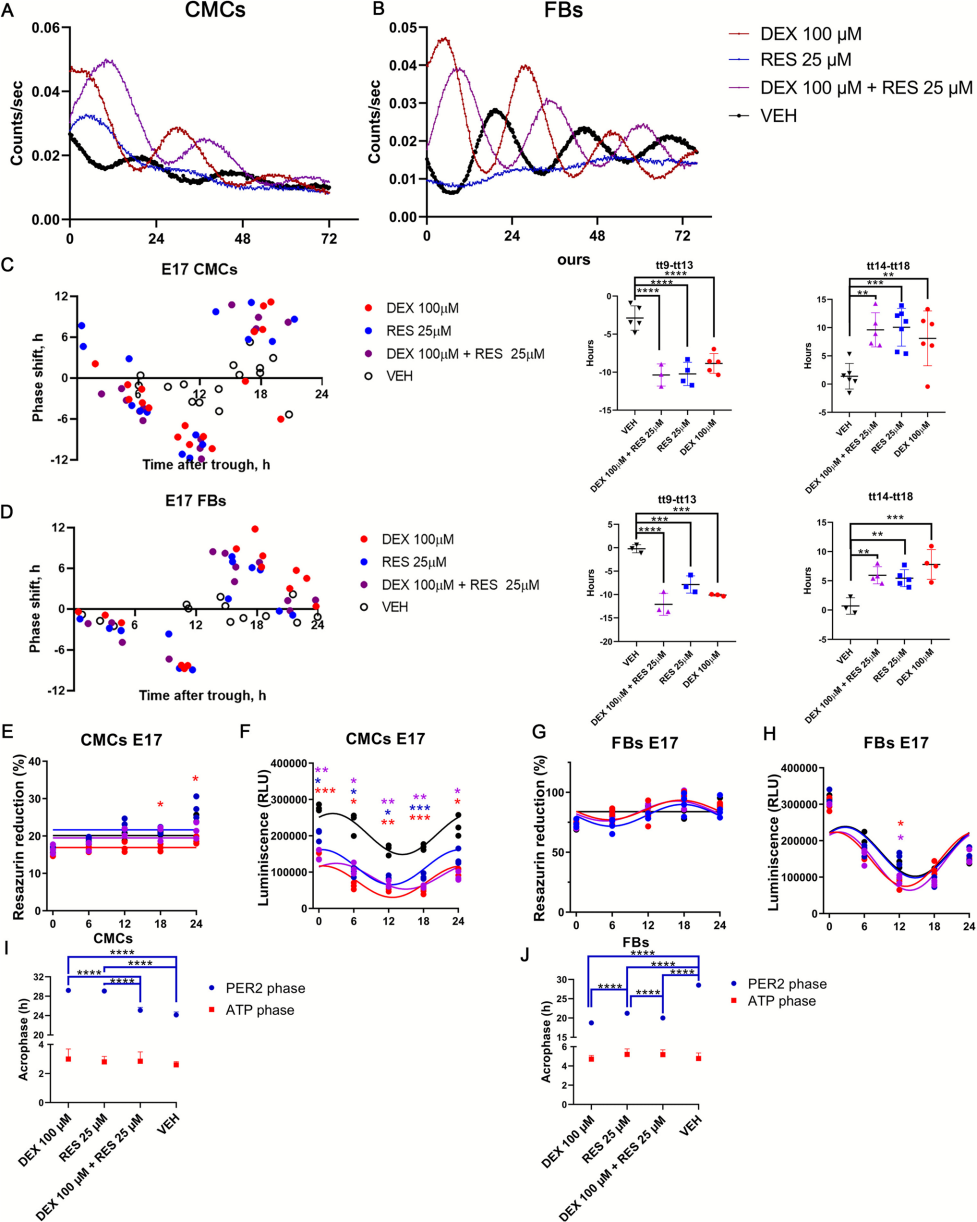
Treatment with RES and DEX resulted in a circadian but not ATP phase shift in CMCs and FBs. For these experiments, at least seven embryonic hearts from six litters were pooled with subsequent isolation of CMCs and FBs. Cells were seeded at 60,000 cells/100 μl for all experiments. Bioluminescence recordings from *mPer2*^*Luc*^ E17 CMCs **(A)** and FBs **(B)** after DEX and RES treatment. PRC for DEX (red), RES (blue), DEX + RES (purple) and VEH (black) treatments of CMCs (*n* = 66) **(C)** and FBs (*n* = 55) **(D)**. Data for each treatment are displayed as the extent of phase shift (+ advance, − delay) resulting from treatment administered at the specific time relative to the bioluminescence peak (indicated as time 12 h). In addition, treatment groups were binned into two intervals (tt9 – tt13 and tt14 - tt18) to compare changes in PRCs. Data were compared using 1-way ANOVA with Dunnett's multiple comparisons. Asterisks indicate the results of Dunnett's multiple comparisons test between treatment groups. Next, to study the rhythm of mitochondrial function, we performed an ATP luminescent assay at five different time points in CMCs **(E)** and FBs **(G)**. Cells were isolated from three separate litters, where embryonic hearts from at least seven fetuses were pooled together. We used an ATP assay to further study the rhythmic change of mitochondrial function in CMCs **(F)** and FBs **(H)** with subsequent cosinor analysis. The ATP and resazurin reduction levels were compared between experimental groups by 2-way ANOVA with Tukey’s multiple comparison tests to the VEH group. The acrophase of CMCs (**I**) and FBs (**J**) ATP and PER2 rhythm was compared using 2-way ANOVA with Tukey’s multiple comparison tests. ∗*P* < 0.05, ∗∗*P* < 0.005, ∗∗∗*P* < 0.0005, ∗∗∗∗*P* < 0.0001

The application of DEX and RES had significant effects on the phases of the PER2-bioluminescence traces in CMCs (Fig. 3A) and FBs (Fig. 3B). Compared to VEH, the most pronounced effects were observed for 100 μM DEX and 100 μM DEX + 25 μM RES. To construct the full phase response curves (PRCs) for 100 μM DEX, 25 μM RES, and their co-treatment compared to VEH, we treated the CMC and FB cultures with them at various time points over 24 h and analyzed the resulting phase shifts of the PER2-driven bioluminescence rhythm (Fig. 3C and 3D, left graphs). The treatment time (tt) 0 and 12 h represent the trough and peak of the bioluminescence rhythm, respectively (Supplementary Table 2). We found that when treated individually, DEX and RES induced similar phase shifts, the magnitudes of which were highly dependent on the timing of the treatment, whereas VEH led to only minor changes in phases in both CMCs (−0.58 ± 2.95 h, *n* = 15) and FBs (−0.42 ± 2.12 h, *n* = 14). DEX + RES treatment resulted in similar PRCs as DEX and RES; the tt12 (PER2 peak) was the transition point between the delays and advances in both CMCs (Fig. 3C) and FBs (Fig. 3D). The maximal phase shift was observed during the time intervals tt9–tt13 and tt14–tt18, where the phase changes were most dramatic in DEX and RES experimental groups for both cell types. In particular, treatment between tt9 – tt13 resulted in a significant phase delay and treatment between tt14 - tt18 led to a phase advance. Comparison of the binned PRC for the VEH group with the rest of the treatment groups (DEX, RES and DEX + RES) confirmed significant effects of these treatments (1-way ANOVA with Dunnett's multiple comparisons, Fig. 3C and 3D, right graphs). These results provide evidence that DEX, RES and their co-treatment entrain the circadian clock in the fetal heart in a similar way.

Next, we compared the observed effects of DEX, RES and DEX+RES on the circadian clock with their effects on the circadian rhythms of energy metabolism in the CMCs and FBs. To assess the rhythm in mitochondrial function, we performed a resazurin assay that is based on the detection of the activity of NADPH dehydrogenase [16] and the availability of NADH, NADPH, FMNH, FADH and cytochromes in the cytosol (reviewed in [40]). A shallow rhythm of resazurin reduction was observed in FBs (cosinor analysis; 100 μM DEX, *P* = 0.0275; 25 μM RES, *P* = 0.0093; 100 μM DEX + 25 μM RES, *P* = 0.0147; VEH, *P* = 0.198), but no significant differences in resazurin reduction rates were observed between the groups (2-way ANOVA with Tukey’s multiple comparison tests; Fig. 3G). In CMCs, no circadian rhythm in resazurin reduction was detected (cosinor analysis; 100 μM DEX, *P* = 0.869; 25 μM RES, *P* = 0.784; 100 μM DEX + 25 μM RES, *P* = 0.124; VEH, *P* = 0.649) and levels of the parameter were significantly lower at 18 and 24 h after DEX treatment (2-way ANOVA with Tukey’s multiple comparison tests; Fig. 3E). Next, we detected ATP levels in samples collected in 6 h intervals over 24 h cycle (0, 6, 12, 18, and 24) after synchronization using an ATP luminescent kit. The results showed a clear rhythm in ATP levels in both CMCs (cosinor analysis; Supplementary Table 2; Fig. 3F) and FBs (cosinor analysis; Supplementary Table 2; VEH, *P* = 0.0434; Fig. 3H). Additionally, using 2-way ANOVA with Tukey’s multiple comparison test, we observed a decrease in ATP level in 100 μM DEX, 25 μM RES and DEX+RES groups in comparison to VEH across all the time points in CMCs (Fig. 3F, Supplementary Table 2). In FBs, downregulation of ATP level was found only at one time point (12 h) in 100 μM DEX (DEX 100 μM vs. VEH, *P* = 0.0433; Fig. 3H) and 100 μM DEX + 25 μM RES groups (Dex 100 μM + RES 25 μM vs. VEH, *P* = 0.0352; Fig. 3H).

For further investigation of the effect of RES and DEX on fetal cardiac cells, we analyzed the expression of *Bcl2*, which is involved in the regulation of the mitochondrial outer membrane permeability and *Birc5* gene as a marker of mitochondrial metabolism by RT-qPCR in the CMCs and FBs after 24 h of treatment with 100 μM DEX, 25 μM RES, co-treatment, and VEH. The relative expression of *Bcl2* was downregulated in the DEX-treated and DEX + RES co-treated groups in CMCs (1-way ANOVA with Dunnett's multiple comparisons test; 100 μM DEX vs. VEH, *P* = 0.0357; 100 μM DEX + 25 μM RES vs. VEH, *P* = 0.0161; Supplementary Fig. 4A). In FBs, *Birc5* expression was decreased after DEX treatment and its co-treatment with RES (VEH vs. 100 μM DEX, *P* = 0.0001; VEH vs. 100 μM DEX + 25 μM RES, *P* = 0.0006; Supplementary Fig. 4B; Supplementary Table 2). The results showed the presence of rhythm of mitochondrial function in the form of ATP and NADH rhythmicity in FBs and a rhythm of ATP level in CMCs, where all treatments had a negative effect on mitochondrial metabolism. Also, our data showed an aggravation of mitochondrial energy metabolism 24 h after DEX and RES treatments in CMCs. In FBs, DEX led to a mild impairment of mitochondrial homeostasis due to downregulation of *Birc5*, which was not accompanied by the ATP content.

Because measurement of ATP and PER2-driven bioluminescence in our samples shares exactly the same experimental design and was performed under the same experimental conditions, we compared the effects of 25 μM RES, 100 μM DEX and RES+DEX on the phases of the PER2 and ATP rhythms by assessing their acrophases using cosinor analysis. Statistical evaluation was done using 2-way ANOVA with Tukey’s multiple comparison tests within each experiment (PER2 bioluminescence or ATP). Interestingly, the acrophases of PER2-driven bioluminescence in both CMCs and FBs were significantly affected by the treatments (*P* < 0.0001, Fig. 3I and J, respectively) but acrophases of ATP rhythm did not show any difference between groups in CMCs (100 μM DEX vs. VEH, *P* = 0.999; 25 μM RES vs. VEH, P > 0.999; 100 μM DEX + 25 μM RES vs. VEH, P > 0.999; Fig. 3I) and FBs (100 μM DEX vs. VEH, P > 0.999; 25 μM RES vs. VEH, *P* = 0.983; 100 μM DEX + 25 μM RES vs. VEH, *P* = 0.985; Fig. 3J).

Overall, the results provide the first evidence for a possible decoupling between the molecular clock and the rhythm of cellular ATP content in the fetal heart.

### Mitochondrial network morphology, membrane potential, and ROS in fetal heart FBs exhibit a circadian rhythm that is not shifted by DEX and RES

**Fig. 4:**
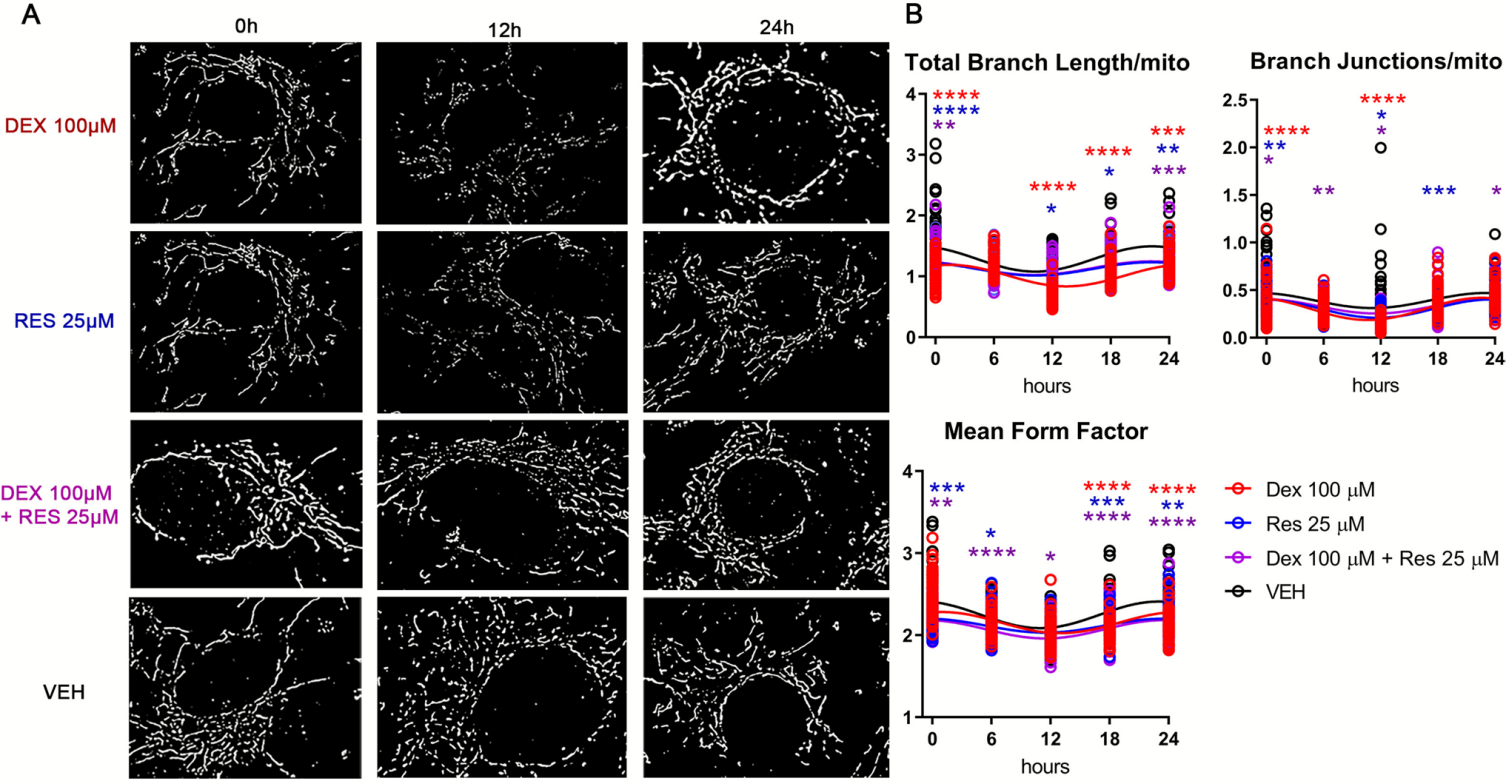
The rhythm of mitochondrial network morphology is synchronized in all treatment groups in FBs. Cells were isolated from the pooled murine male and female fetal hearts from three litters and seeded at 60,000 cells/100 μl. After the synchronization and beginning of the treatment with DEX, RES, co-treatment or VEH, E17 FBs were incubated with MitoTracker Red at a certain time point. Images were acquired using a Leica SP8 confocal microscope (**A**). Images were analyzed using the Mitochondria Analyzer ImageJ plugin. The total branch length, branch junction and mean form factor parameters were used to analyze the rhythm of mitochondrial dynamics (**B**). The data were analyzed using cosinor analysis and 2-way mixed ANOVA analysis with Tukey’s multiple comparison tests. ∗*P* < 0.05, ∗∗*P* < 0.005, ∗∗∗*P* < 0.0005, ∗∗∗∗*P* < 0.0001

To further investigate the rhythm in mitochondrial function in the fetal heart and its responses to 25 μM RES, 100 μM DEX and RES+DEX, we assessed the daily profiles of changes in mitochondrial network morphology using Mitotracker Red (Fig. 4A) in fetal FBs, which exhibited robust ATP and NADH rhythmicity. We utilized the Mitochondria Analyzer ImageJ plugin to quantify changes in the mitochondrial network, which is a powerful tool for multiplexed analysis of mitochondrial state and function [12]. We assessed parameters such as total branch length, which represents the sum of the length of all branches in a cell’s mitochondria, branch junctions/mito (the number of junctions per mitochondrion) and the mean form factor reflects the shape of mitochondria, where value 1 indicates round mitochondria. Using cosinor analysis, we confirmed the presence of synchronized mitochondrial dynamics in all treated groups using such parameters as total branch length, branch junction, and mean form factor (cosinor analysis; Supplementary Table 2; Fig. 4B). According to cosinor analysis, the lowest form factor score was at the treatment time 12 (tt12) in all treated groups. In addition, both RES and DEX treatment led to a decrease of the values of all analyzed parameters at all time points (Supplementary Table 2, Fig. 4B).

**Fig. 5:**
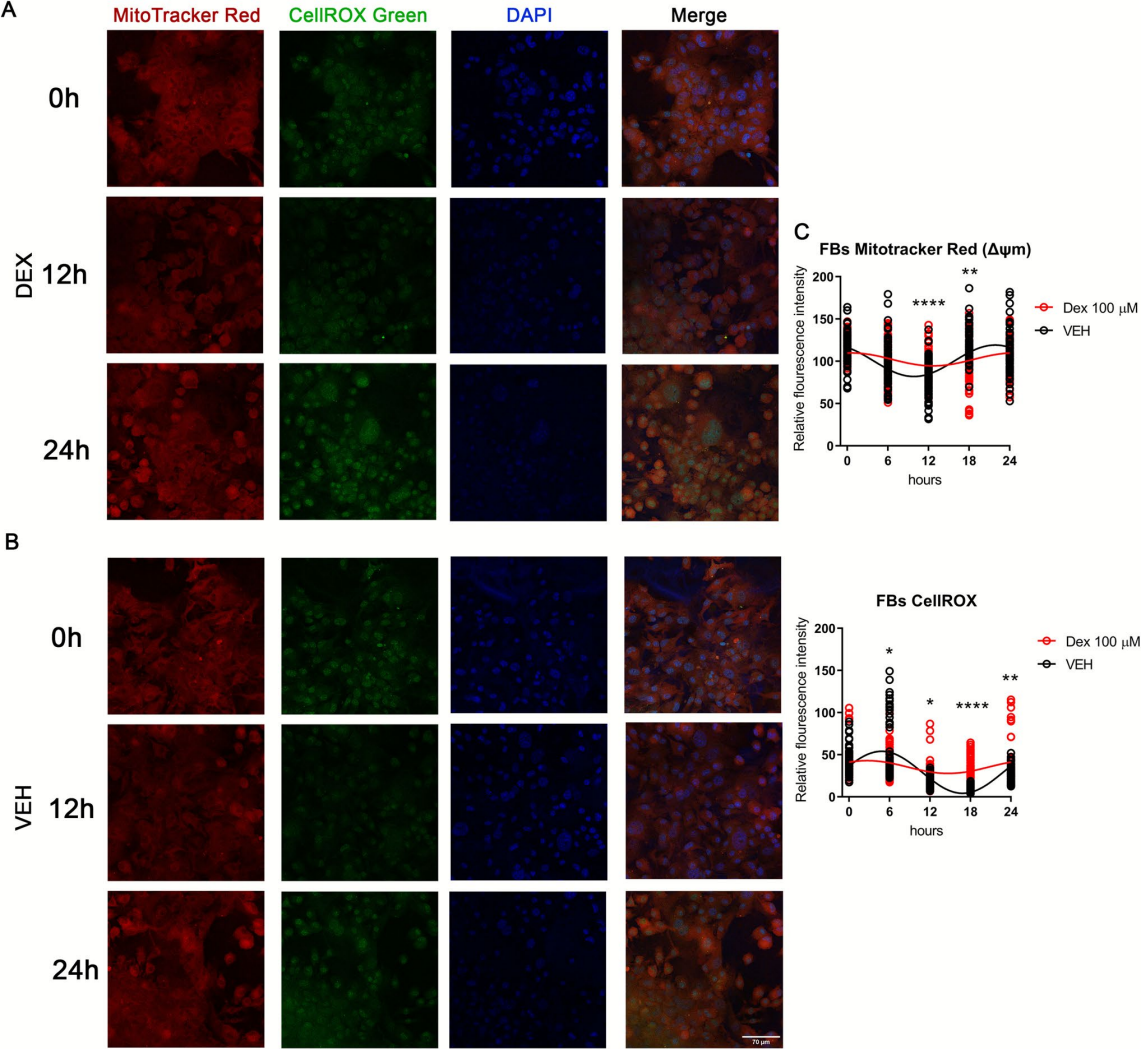
Mitochondrial membrane potential (MMP) and reactive oxygen species (ROS) displayed synchronized rhythm in fetal heart FBs. E17 FBs were isolated from the pooled murine male and female fetal hearts from three litters, seeded at 60,000 cells/100 μl, and synchronized using serum shock with further treatment with 100 μM DEX (**A**) or VEH (**B**). The incubation with the MitoTracker Red and CellROX Green reagents was started at five different time points, 6 h apart. Images were acquired using a Leica SP8 confocal microscope. The images were later analyzed in the ImageJ software by measuring the mean intensity in the ROI. The statistical evaluation was done using cosinor analysis in the GraphPad Prism software and showed the presence of synchronized rhythm in FBs after DEX and VEH treatments (**C**). In addition, the data were analyzed using 2-way mixed ANOVA analysis with Sidak’s multiple comparison tests. ∗*P* < 0.05, ∗∗*P* < 0.005, ∗∗∗*P* < 0.0005, ∗∗∗∗*P* < 0.0001. Scale bar – 70 μm

Next, we compared daily profiles of mitochondrial membrane potential (MMP) and reactive oxygen species (ROS) in samples treated with VEH and 100 μM DEX, which had the most prominent effect on mitochondrial function in the fetal FBs. Cosinor analysis confirmed circadian rhythm in the presence of ROS and MMP, both after DEX (Fig. 5A) and VEH (Fig. 5B). The analysis showed synchronized rhythm of MMP and ROS (cosinor analysis; Supplementary Table 2; Fig. 5C). The trough of FBs MMP was at tt12 for both treatments. For ROS, the trough was detected at tt18 for both DEX and VEH groups, although the rhythm was considerably shallower in the DEX group compared to the VEH group. Also, 100 μM DEX treatment led to the reduction of the amplitude of the MMP and ROS rhythms (Supplementary Table 2, Fig. 5C).

Overall, our results demonstrated the presence of synchronized rhythms of mitochondrial morphology, MMP and ROS production in the fetal FBs, where RES, DEX and their co-treatment resulted in a decrease in parameters of mitochondrial function.

## Discussion

Here we show that RES affects the circadian clocks differently in two types of fetal heart cells and can modulate the effect of DEX on the clocks in these cells. RES exhibits a dose-dependent effect on PER2 stability in both cell types. In CMCs, RES modulates the half-life of PER2 at least in part via the adenylyl cyclase pathway. Considering the role of the clock in regulating mitochondrial function, we further investigated whether there is rhythmic mitochondrial activity after treatment with DEX and RES. Our results show a rhythmic pattern of ATP production in both cell types. Particularly in FBs of the fetal heart, we provide the first evidence for a possible decoupling of mitochondrial rhythmicity from the circadian clock, as evidenced by synchronized rhythms of mitochondrial dynamics and bioenergetics in all treatment groups.

RES can affect the circadian clock via multiple pathways. For example, the clock is modulated by changes in the acetylation state of the clock proteins PER2 and BMAL1 via histone deacetylase SIRT1 [59], which is activated by RES [44, 61]. SIRT1 uses a NAD+-dependent mechanism for deacetylation of histone and non-histone targets that eventually acts as a regulator of numerous downstream metabolic and physiological processes [14]. Also, the SIRT1-mediated PER2 deacetylation promotes its subsequent degradation [2, 26]. It was also shown that SIRT1 could modulate BMAL1 in a similar manner [54]. According to that study, SIRT1 removes the acetyl group from Lys537 of BMAL1, which increases the repression efficiency of CRY to inhibit CLOCK::BMAL1-mediated transcription. In addition, upon activation, SIRT1 might downregulate *Per1* expression via interaction with the CLOCK/BMAL1 complex [59]. The effect of RES on the clock is well documented in many cell types and tissues (reviewed in [69]).

Previously, it has been shown that RES decreases the amplitude of *Bmal1* mRNA in C2C12 myotubes [4]. Interestingly, it was shown that the effect of RES is age-specific. Human young lung fibroblast cells treated with 100 μM RES displayed upregulation of *Bmal1* mRNA level and downregulation of *Per1* level. However, both *Bmal1* and *Per1* mRNA levels were upregulated after the same treatment in human old lung fibroblast cells [57]. Also, RES was reported to modulate the circadian expression of several clock genes in human adipose progenitor cells [38]. Nevertheless, the effect of RES on the circadian clock in the fetal heart has not been well studied. Our data show that RES has a strong dose-dependent effect on the period and phase of the circadian clock in the fetal CMCs and FBs. Whereas the lower dose of RES (5 μM) did not significantly affect PER2 bioluminescence alone or in co-treatment with DEX, its higher dose (25 μM) lengthened the period in CMCs but shortened the period in FBs. Interestingly, DEX treatment alone led to cell-type-dependent changes in the period, with period prolongation in CMCs and period shortening in FBs. Moreover, both DEX and RES (25 μM) shortened the period of the circadian clock in FBs compared to VEH, but their co-treatment prevented the change in the period. RES (25 μM) prevented DEX-induced effects on the increase in amplitude in FBs. Conversely, DEX-induced changes of amplitude and period were not mitigated by RES in CMCs. Previous studies suggested a mechanism of this dose-dependency in the human umbilical vein smooth muscle cells [77]; the low-dose RES (3 μM) activated AKT via SIRT1, and a high-dose RES (30 μM) resulted in AMPK-mediated mTORC1 inhibition.

It was shown that RES-induced SIRT1 overexpression decreased PER2 stability [2, 45]. In addition, SIRT1 can also influence the subcellular localization of PER2, because inhibition of SIRT1 by EX-527 resulted in a significant increase in PER2 cytoplasmic levels [3]. Unexpectedly, in this study, we show that in the fetal CMCs and FBs, RES stabilize PER2 via prolonging PER2 half-life in a dose-dependent manner. Interestingly, the effect on PER2 stability was accompanied by the shortening of the period in FBs, which suggests an additional effect of RES on other parts of the TTFL. One possible mechanism of the increase of PER2 stability after RES treatment could involve SIRT6, because 20 μM RES increased expression of SIRT6 [58] and deacetylation of PER2 by SIRT6 was found to increase its stability in HEK293 cells [72]. In addition, RES led to upregulation of *Sirt1* and downregulation of *Sirt6* in human young lung fibroblast cells [57], but the opposite picture was observed upon the same treatment in human old lung fibroblast cells. However, we cannot support the involvement of SIRT6 in stabilizing PER2 by RES in our cells, because inhibition of SIRT6 by OSS_128167 did not result in the shortening of the PER2 half-life in both CMCs and FBs. Previously, Eckle et al. [22] showed that proteasomal degradation of PER2 can be inhibited in an adenosine receptor A2b (ADORA2b)-dependent manner. The stability of PER2, in this case, was increased due to the activation of the cAMP/CREB pathway via ADORA2b signaling. Additionally, RES was shown to act as an agonist of the cAMP signaling pathway [63]. Here we found that the effect of RES on PER2 stabilization in CMCs but not in FBs can be partially prevented via inhibition of adenylyl cyclase by treatment with MDL-12,330A hydrochloride.

In addition, treatment with 25 μM RES and 100 μM DEX resulted in a significant decrease in ATP levels in CMCs but not in FBs. The reduction in mitochondrial metabolism was further confirmed by RT-qPCR analyses of *Bcl2*, which is involved in the function of the mitochondrial permeability transition pore complex (reviewed in [86]), and *Birc5* mRNA, which is associated with the regulation of mitochondrial metabolism, ROS accumulation and mitochondrial fission [29]. We confirmed the presence of DEX- and RES-induced impairment of ATP metabolism in CMCs, but not in FBs. Nevertheless, mitochondrial metabolism might also be downregulated in FBs due to the decrease in *Birc5* level. Interestingly, the downregulation of ATP content in CMCs might be, at least partially, explained by the downregulation of *Bcl2*, which has a potential role in the regulation of ATP level [49]. These findings demonstrate the cell-dependent response to DEX and RES treatment in terms of mitochondrial metabolism.

Prenatal DEX application influences the development and maturation of many organs such as the heart, kidney and brain (reviewed in [56]). It was also shown that DEX accelerates the development of the central pacemaker SCN in the fetal stage and entrains the circadian rhythm of the fetal SCN in vitro [9]. However, its effect on the circadian clock in the fetal heart has not been studied. On the molecular level, glucocorticoids influence the clock components in multiple ways. For example, glucocorticoids increase the expression of *Per2* by binding to its intronic sequence in mouse primary marrow stromal cells [68]. Meanwhile, glucocorticoid-induced transcriptional repression of *Rev-Erbα* and *Rorα* genes is achieved by the presence of negative glucocorticoid response elements in their promoters [73]. Here, we show that DEX treatment (100, 10 or 1 μM) leads to the increase in amplitude of the PER2 bioluminescence rhythm in primary CMC and FB cultures, in accordance with a similar effect in the atrium of the adult heart of *mPer2*^*Luc*^ mouse [79]. In addition, we show that DEX in all tested concentrations shortened the PER2 period in FBs but prolonged in CMCs. Apparently, the effect of glucocorticoids on period has a cell-type-specificity, because period of PER2 bioluminescence rhythm was prolonged after application of 100 nM DEX in murine hippocampus explants [48] and 1 μM DEX in mouse embryonic fibroblasts [13].

The effects of both DEX and RES on the phase of the circadian clock were previously well established in various cells, including the heart, but not on the adult master clock in the SCN [5]. During the fetal stage, DEX also shifts the SCN clock of *mPer2*^*Luc*^ mice [9]. DEX induces either phase advances or delays depending on the time of application *in vitro* in mouse embryonic fibroblasts [13], lung and kidney [43], nasal mucosa [33], heart tissues [84] and using *in silico* models [32]. The effects of RES treatments on the phase of the clock have also been documented. Treatment of the alpha-mouse-liver-12 cell line with 50 μM RES resulted in phase advance with decreased amplitude of *Bmal1* mRNA oscillation [11]. In this study, we present data that are in accordance with the previous studies. We performed treatments over a full circadian cycle and showed that both DEX and RES treatments shift the phase of the clock in the fetal CMCs and FBs dramatically in a time-dependent manner. Our data identified the most sensitive windows during the circadian cycle when the fetal CMCs and FBs clocks are most sensitive to DEX and RES treatments; they both induced the maximal phase delays during tt9 – tt13 and the maximal phase advances during tt14 - tt18, which were significantly larger than the VEH-induced responses.

Next, we aimed to analyze the interconnection between the circadian clock and energy metabolism in the fetal heart cell cultures. It has been shown that the mature circadian clock tightly controls energy and oxidative metabolism (reviewed in [64]). However, this link has not been fully studied for fetal cells. Genetic deletion of *Per2* did not result in significant downregulation of the number of mitochondria or mtDNA copy numbers in MEF [50]. Mitochondrial fusion (dynamin-related protein 1) and fission (mitofusin 2) proteins, together with the redox state of mitochondria in the embryonic avian retina and 661 W photoreceptor cell line displayed circadian rhythm, respectively [10]. Moreover, only dynamin-related protein 1 was under circadian control in both cell lines. Deletion of *Bmal1* resulted in upregulated mRNA expression of genes related to mitochondrial complex I–V and the tricarboxylic acid cycle in embryonic stem cells [1]. Additionally, *Bmal1* deletion led to the metabolic switch with translation into a more oxidative versus glycolytic use of glucose. Chemical energy produced by mitochondria is stored in the form of an adenosine triphosphate (ATP), and ATP content exhibits a diurnal rhythm in the SCN and retina [35, 83]. However, there is no data available on circadian ATP rhythms in fetal CMCs and FBs. Here we demonstrate the presence of the ATP rhythm in E17 CMCs and FBs in the 100 μM DEX, 25 μM RES, their co-treatment, and VEH-treated groups. Mitochondrial function analyzed in models such as skeletal muscle [80], heart tissue [41] and myoblast cell culture [37] was previously firmly linked to the circadian clock. However, comparison of the acrophases of the ATP and PER2-driven bioluminescence in FBs revealed that their treatment-induced phases are not synchronized. Therefore, we observed synchronized rhythm of the ATP level in cell cultures after DEX or RES treatment. To verify the result, we used additional markers of oxidative metabolism – resazurin assay (NADH levels), mitochondrial morphology, membrane potential and reactive oxygen species, focusing mostly on fetal heart FBs. All four methods showed presence of circadian rhythms of selected markers in FBs. Importantly, the circadian phase parameter of these markers was insensitive to DEX and RES application, similar to ATP levels and in contrast to PER2 rhythms. These observations suggest at least a partially independent mechanism between TTFL and the metabolic rhythms (reviewed in [70]). Along with our findings, it was recently shown that markers of mitochondrial function and oxidative metabolism do not display circadian regulation in adult murine skeletal muscle [24]. Overall, this finding is the first evidence of possible uncoupling of the rhythm of mitochondrial dynamics and function from the circadian clock under specific conditions in the fetal heart.

Our study has some limitations. Since the amplitude of TTFL and the metabolic clock are markedly different, it is possible that our methods were unable to detect subtle changes in phase that are readily visible when using the PER2 luminescence marker. In addition, we do not provide information on the molecular mechanism of the effect of RES on DEX-induced changes of PER2-driven bioluminescence period and amplitude.

Overall, our study sheds light on the non-canonical effect of RES in the fetal heart cells, which prolongs PER2 half-life partially via the adenylyl cyclase pathway in CMCs. We also showed that RES has a cell-specific effect on the clock in the fetal heart; it inhibits the effects of DEX on clock in FBs but not CMCs. In CMCs and FBs, RES had an effect on PER2-driven bioluminescence parameters in a dose-dependent and cell-type-dependent manner. In particular, we observed the opposite effect on the period after both DEX and RES application in CMCs and FBs. Our study provides the first evidence that in fetal heart FBs, the rhythm of mitochondrial dynamics and bioenergetics can be uncoupled from the clock upon DEX and RES treatment. These results may advance our knowledge of the interconnection between the clock and mitochondrial rhythm during the fetal stage.

## Supplementary Information

ESM 1 (DOCX 12 kb)

ESM 2 (DOCX 37 kb)

ESM 3 Supplementary Fig. 1. Dose-dependent effect of RES on oscillatory parameters in FBs and CMCs. Isolated cells were seeded at 60,000 cells/100 μl and, after synchronization, were treated with DEX (100, 10 or 1 μM), co-treated with RES (5 or 25 μM), and VEH. The bioluminescence parameters alterations were evaluated by comparing the period, amplitude, decay, and trend of the experimental groups in CMCs (**A**) and FBs (**B**). Cells were isolated from pooled seven or more fetal hearts from each of six pregnant mice. Data were compared using 1-way ANOVA with Tukey’s multiple comparison test and presented as individual ratios and mean ± SD. Asterisks show the results of Tukey's multiple comparisons test between treatment groups. ∗*P* < 0.05, ∗∗*P* < 0.005, ∗∗∗*P* < 0.0005, ∗∗∗∗*P* < 0.0001. (PNG 721 kb)

ESM 4 Supplementary Fig. 2. RES prolongs PER2 half-life in a dose-dependent manner. Cells were isolated from at least seven fetuses from each of three pregnant mice and seeded at 60,000 cells/100 μl. CMCs (**A**) and FBs (**B**) were treated with 5, 25 and 50 μM RES or VEH. Treatment with cycloheximide (40 μg/ml) was done after reaching peak of bioluminescence with subsequent recording of PER2 degradation curve (**A** and **B**, left graphs). Half-life was analysed using 1-way ANOVA with Tukey multiple comparisons test (**A** and **B**, right graphs). ∗*P* < 0.05, ∗∗*P* < 0.005, ∗∗∗*P* < 0.0005, ∗∗∗∗*P* < 0.0001. (PNG 221 kb)

ESM 5 Supplementary Fig. 3. SIRT6 inhibitor (SIRT6i) did not reduce RES-increased PER2 half-life. CMCs and FBs were isolated from at least seven fetuses from each of three pregnant mice. Cells were seeded at 60,000 cells/100 μl and subsequently treated with 25 μM RES, co-treated with 150 μM SIRT6i (OSS_128167) or VEH. Upon reaching a peak of the bioluminescence, cells were treated with cycloheximide (40 μg/ml) following recording of PER2 degradation curve in CMCs and FBs (**A** and **B**, left graphs). PER2 half-life was quantified by fitting exponential decay curves to the first 8 h of luminescence data post-CHX treatment. Half-life was analysed using 1-way ANOVA with Tukey multiple comparisons test (**A** and **B**, right graphs). ∗*P* < 0.05, ∗∗*P* < 0.005, ∗∗∗*P* < 0.0005, ∗∗∗∗*P* < 0.0001. (PNG 299 kb)

ESM 6 Supplementary Fig. 4. The level of markers of mitochondrial function was downregulated after DEX application in CMCs and FBs. To study the effect of RES and DEX treatments on mitochondrial metabolism, RT-qPCR assay was done to detect the level of Bcl2 and Birc5 genes in CMCs (C) and FBs (D). Cells were isolated from pooled seven or more fetal hearts from each of four pregnant mice. Both CMCs and FBs were seeded at 60,000 cells/100 μl. Statistics were analyzed by 1-way ANOVA with Dunnett's multiple comparisons test to VEH. ∗*P* < 0.05, ∗∗*P* < 0.005, ∗∗∗*P* < 0.0005, ∗∗∗∗*P* < 0.0001. (PNG 134 kb)

## Data Availability

The data used to support the findings of this study are available from the corresponding author upon reasonable request.
