# Supplementary material for: Resveratrol and dexamethasone have cell-specific effects on the circadian clock but not on the rhythm of mitochondrial function in the fetal heart
Source: J Physiol Biochem. 2026 Feb 16;82(1):11. doi: 10.1007/s13105-026-01152-8 (PMC12909427; doi:10.1007/s13105-026-01152-8)
Supplement: Supplementary file 1 — (DOCX 12 kb) [file 13105_2026_1152_MOESM1_ESM.docx]

Supplementary Table S1. List of forward and reverse primers used for gene expression analysis.

| Primer | Forward | Reverse | Predesigned, manufactured | Conc. (µM) |
| --- | --- | --- | --- | --- |
| *Gapdh* | TGTCCGTCGTGGATCTGAC | CCTGCTTCACCACCTTCTTG | KicqStart (Merck) | 0.3 |
| *Hprt1* | AGGGATTTGAATCACGTTTG | TTTACTGGCAACATCAACAG | KicqStart (Merck) | 0.3 |
| *Pgk1* | CTATCATAGGTGGTGGAGAC | ACACTAGGTTGACTTAGGAG | KicqStart (Merck) | 0.3 |
| *Bcl2* | ATGACTGAGTACCTGAACC | ATATAGTTCCACAAAGGCATC | KicqStart (Merck) | 0.3 |
| *Birc5* | TAGAGGAGCATAGAAAGCAC | CTCTTTTTGCTTGTTGTTGG | KicqStart (Merck) | 0.3 |
