# Supplementary material for: Resveratrol and dexamethasone have cell-specific effects on the circadian clock but not on the rhythm of mitochondrial function in the fetal heart
Source: J Physiol Biochem. 2026 Feb 16;82(1):11. doi: 10.1007/s13105-026-01152-8 (PMC12909427; doi:10.1007/s13105-026-01152-8)
Supplement: Supplementary file 2 — (DOCX 37 kb) [file 13105_2026_1152_MOESM2_ESM.docx]

**Supplementary Table 2.** Absolute and P values of the bioluminescence recording and PER2 decay experiments.

1. **RES 25 μM but not 5 μM prevents the effect of DEX on the rhythm in PER2::LUC bioluminescence in CMCs and FBs (Fig. 1)**

| **Cell type** | **Parameter** | **Groups** | **Values** | **P value** | **Statistical method** | **Figure** |
| --- | --- | --- | --- | --- | --- | --- |
| CMCs | Period | 100 μM DEX vs. VEH | 26.28 ± 1.12 h vs. 24.94 ± 1.22 h | P = 0.0477 | 1-way ANOVA with Tukey’s multiple comparison test | Fig. 1A |
| CMCs | Period | 100 μM DEX vs. DEX 100 µM + RES 25 µM | 26.28 ± 1.12 h vs. 28.12 ± 1.14 h | P = 0.0006 | 1-way ANOVA with Tukey’s multiple comparison test | Fig. 1A |
| CMCs | Period | 10 μM DEX vs. DEX 10 µM + RES 25 µM | 25.14 ± 0.44 h vs. 28.21 ± 1.31 h | P < 0.0001 | 1-way ANOVA with Tukey’s multiple comparison test | Fig. 1A |
| CMCs | Period | 1 μM DEX vs. DEX 1 µM + RES 25 µM | 25.64 ± 1.28 h vs. 27.7 ± 1.99 h | P = 0.0173 | 1-way ANOVA with Tukey’s multiple comparison test | Fig. 1A |
| CMCs | Period | DEX 100 µM + RES 25 µM vs. VEH | 28.12 ± 1.14 h vs. 24.94 ± 1.22 h | P < 0.0001 | 1-way ANOVA with Tukey’s multiple comparison test | Fig. 1A |
| CMCs | Period | DEX 10 µM + RES 25 µM vs. VEH | 28.21 ± 1.31 h vs. 24.94 ± 1.22 h | P < 0.0001 | 1-way ANOVA with Tukey’s multiple comparison test | Fig. 1A |
| CMCs | Period | DEX 1 µM + RES 25 µM vs. VEH | 27.7 ± 1.99 h vs. 24.94 ± 1.22 h | P < 0.0001 | 1-way ANOVA with Tukey’s multiple comparison test | Fig. 1A |
| CMCs | Period | 5 μM RES vs. VEH | 24.8 ± 1.23 h vs. 24.94 ± 1.22 h | P > 0.9999 | 1-way ANOVA with Tukey’s multiple comparison test | Fig. 1A |
| CMCs | Period | 25 μM RES vs. VEH | 26.7 ± 2.13 h vs. 24.94 ± 1.22 h | P = 0.0042 | 1-way ANOVA with Tukey’s multiple comparison test | Fig. 1A |
| FBs | Period | 100 μM DEX vs. VEH | 24.48 ± 0.6 h vs. 25.24 ± 0.65 h | P = 0.0001 | 1-way ANOVA with Tukey’s multiple comparison test | Fig. 1B |
| FBs | Period | 10 μM DEX vs. VEH | 24.53 ± 0.45 h vs. 25.24 ± 0.65 h | P = 0.0006 | 1-way ANOVA with Tukey’s multiple comparison test | Fig. 1B |
| FBs | Period | 1 μM DEX vs. VEH | 24.53 ± 0.51 h vs. 25.24 ± 0.65 h | P = 0.0005 | 1-way ANOVA with Tukey’s multiple comparison test | Fig. 1B |
| FBs | Period | 100 μM DEX vs. DEX 100 µM + RES 25 µM | 24.48 ± 0.6 h vs. 25.14 ± 0.62 h | P = 0.0434 | 1-way ANOVA with Tukey’s multiple comparison test | Fig. 1B |
| FBs | Period | 10 μM DEX vs. DEX 10 µM + RES 25 µM | 24.53 ± 0.45 h vs. 25.21 ± 0.59 h | P = 0.0211 | 1-way ANOVA with Tukey’s multiple comparison test | Fig. 1B |
| FBs | Period | 5 μM RES vs VEH | 25.5 ± 0.87 h vs. 25.24 ± 0.65 h | P = 0.9369 | 1-way ANOVA with Tukey’s multiple comparison test | Fig. 1B |
| FBs | Period | 25 μM RES vs. VEH | 24.3 ± 0.88 h vs. 25.24 ± 0.65 h | P < 0.0001 | 1-way ANOVA with Tukey’s multiple comparison test | Fig. 1B |
| CMCs | Amplitude | 100 μM DEX vs. VEH | 0.022 ± 0.011 vs. 0.003 ± 0.003 | P < 0.0001 | 1-way ANOVA with Tukey’s multiple comparison test | Fig. 1A |
| CMCs | Amplitude | 10 μM DEX vs. VEH | 0.021 ± 0.008 vs. 0.003 ± 0.003 | P < 0.0001 | 1-way ANOVA with Tukey’s multiple comparison test | Fig. 1A |
| CMCs | Amplitude | 1 μM DEX vs. VEH | 0.021 ± 0.01 vs. 0.003 ± 0.003 | P < 0.0001 | 1-way ANOVA with Tukey’s multiple comparison test | Fig. 1A |
| CMCs | Amplitude | DEX 100 µM + RES 5 µM vs. VEH | 0.016 ± 0.011 vs. 0.003 ± 0.003 | P = 0.0006 | 1-way ANOVA with Tukey’s multiple comparison test | Fig. 1A |
| CMCs | Amplitude | DEX 10 µM + RES 5 µM vs. VEH | 0.020 ± 0.008 vs. 0.003 ± 0.003 | P < 0.0001 | 1-way ANOVA with Tukey’s multiple comparison test | Fig. 1A |
| CMCs | Amplitude | DEX 1 µM + RES 5 µM vs. VEH | 0.019 ± 0.009 vs. 0.003 ± 0.003 | P = 0.0001 | 1-way ANOVA with Tukey’s multiple comparison test | Fig. 1A |
| CMCs | Amplitude | DEX 100 µM + RES 25 µM vs. VEH | 0.018 ± 0.011 vs. 0.003 ± 0.003 | P < 0.0001 | 1-way ANOVA with Tukey’s multiple comparison test | Fig. 1A |
| CMCs | Amplitude | DEX 10 µM + RES 25 µM vs. VEH | 0.022 ± 0.005 vs. 0.003 ± 0.003 | P < 0.0001 | 1-way ANOVA with Tukey’s multiple comparison test | Fig. 1A |
| CMCs | Amplitude | DEX 1 µM + RES 25 µM vs. VEH | 0.02 ± 0.007 vs. 0.003 ± 0.003 | P < 0.0001 | 1-way ANOVA with Tukey’s multiple comparison test | Fig. 1A |
| CMCs | Amplitude | 5 μM RES vs. VEH | 0.003 ± 0.002 vs. 0.003 ± 0.003 | P > 0.9999 | 1-way ANOVA with Tukey’s multiple comparison test | Fig. 1A |
| CMCs | Amplitude | 25 μM RES vs. VEH | 0.003 ± 0.003 vs. 0.003 ± 0.003 | P > 0.9999 | 1-way ANOVA with Tukey’s multiple comparison test | Fig. 1A |
| FBs | Amplitude | 100 μM DEX vs. VEH | 0.038 ± 0.013 vs. 0.010 ± 0.006 | P < 0.0001 | 1-way ANOVA with Tukey’s multiple comparison test | Fig. 1B |
| FBs | Amplitude | 10 μM DEX vs. VEH | 0.03 ± 0.011 vs. 0.010 ± 0.006 | P < 0.0001 | 1-way ANOVA with Tukey’s multiple comparison test | Fig. 1B |
| FBs | Amplitude | 1 μM DEX vs. VEH | 0.031 ± 0.01 vs. 0.010 ± 0.006 | P < 0.0001 | 1-way ANOVA with Tukey’s multiple comparison test | Fig. 1B |
| FBs | Amplitude | DEX 100 µM + RES 5 µM vs. VEH | 0.039 ± 0.016 vs. 0.010 ± 0.006 | P < 0.0001 | 1-way ANOVA with Tukey’s multiple comparison test | Fig. 1B |
| FBs | Amplitude | DEX 10 µM + RES 5 µM vs. VEH | 0.032 ± 0.011 vs. 0.010 ± 0.006 | P < 0.0001 | 1-way ANOVA with Tukey’s multiple comparison test | Fig. 1B |
| FBs | Amplitude | DEX 1 µM + RES 5 µM vs. VEH | 0.032 ± 0.012 vs. 0.010 ± 0.006 | P < 0.0001 | 1-way ANOVA with Tukey’s multiple comparison test | Fig. 1B |
| FBs | Amplitude | 100 μM DEX vs. DEX 100 µM + RES 25 µM | 0.038 ± 0.013 vs. 0.019 ± 0.014 | P < 0.0001 | 1-way ANOVA with Tukey’s multiple comparison test | Fig. 1B |
| FBs | Amplitude | 10 μM DEX vs. DEX 10 µM + RES 25 µM | 0.03 ± 0.011 vs. 0.015 ± 0.011 | P = 0.0002 | 1-way ANOVA with Tukey’s multiple comparison test | Fig. 1B |
| FBs | Amplitude | 1 μM DEX vs. DEX 1 µM + RES 25 µM | 0.031 ± 0.01 vs. 0.006 ± 0.002 | P < 0.0001 | 1-way ANOVA with Tukey’s multiple comparison test | Fig. 1B |
| FBs | Amplitude | 5 μM RES vs VEH | 0.011 ± 0.005 vs. 0.010 ± 0.006 | P > 0.9999 | 1-way ANOVA with Tukey’s multiple comparison test | Fig. 1B |
| FBs | Amplitude | 25 μM RES vs. VEH | 0.008 ± 0.006 vs. 0.010 ± 0.006 | P > 0.9999 | 1-way ANOVA with Tukey’s multiple comparison test | Fig. 1B |
| CMCs | Decay | 100 μM DEX vs. VEH | 0.037 ± 0.012 vs. 0.018 ± 0.011 | P = 0.0005 | 1-way ANOVA with Tukey’s multiple comparison test | Fig. 1A |
| CMCs | Decay | 10 μM DEX vs. VEH | 0.033 ± 0.008 vs. 0.018 ± 0.011 | P = 0.01535 | 1-way ANOVA with Tukey’s multiple comparison test | Fig. 1A |
| CMCs | Decay | 1 μM DEX vs. VEH | 0.036 ± 0.013 vs. 0.018 ± 0.011 | P = 0.01827 | 1-way ANOVA with Tukey’s multiple comparison test | Fig. 1A |
| CMCs | Decay | 1 μM DEX vs. DEX 1 µM + RES 25 µM | 0.036 ± 0.013 vs. 0.053 ± 0.019 | P = 0.0457 | 1-way ANOVA with Tukey’s multiple comparison test | Fig. 1A |
| CMCs | Decay | DEX 100 µM + RES 5 µM vs. VEH | 0.035 ± 0.01 vs. 0.018 ± 0.011 | P = 0.023 | 1-way ANOVA with Tukey’s multiple comparison test | Fig. 1A |
| CMCs | Decay | DEX 10 µM + RES 5 µM vs. VEH | 0.036 ± 0.011 vs. 0.018 ± 0.011 | P = 0.0066 | 1-way ANOVA with Tukey’s multiple comparison test | Fig. 1A |
| CMCs | Decay | DEX 1 µM + RES 5 µM vs. VEH | 0.035 ± 0.012 vs. 0.018 ± 0.011 | P = 0.0166 | 1-way ANOVA with Tukey’s multiple comparison test | Fig. 1A |
| CMCs | Decay | DEX 100 µM + RES 25 µM vs. VEH | 0.037 ± 0.015 vs. 0.018 ± 0.011 | P = 0.0001 | 1-way ANOVA with Tukey’s multiple comparison test | Fig. 1A |
| CMCs | Decay | DEX 10 µM + RES 25 µM vs. VEH | 0.044 ± 0.017 vs. 0.018 ± 0.011 | P < 0.0001 | 1-way ANOVA with Tukey’s multiple comparison test | Fig. 1A |
| CMCs | Decay | DEX 1 µM + RES 25 µM vs. VEH | 0.053 ± 0.019 vs. 0.018 ± 0.011 | P < 0.0001 | 1-way ANOVA with Tukey’s multiple comparison test | Fig. 1A |
| CMCs | Decay | 5 μM RES vs. VEH | 0.016 ± 0.014 vs. 0.018 ± 0.011 | P > 0.9999 | 1-way ANOVA with Tukey’s multiple comparison test | Fig. 1A |
| CMCs | Decay | 25 μM RES vs. VEH | 0.016 ± 0.012 vs. 0.018 ± 0.011 | P > 0.9999 | 1-way ANOVA with Tukey’s multiple comparison test | Fig. 1A |
| FBs | Decay | 100 μM DEX vs. VEH | 0.025 ± 0.004 vs. 0.015 ± 0.009 | P < 0.0001 | 1-way ANOVA with Tukey’s multiple comparison test | Fig. 1B |
| FBs | Decay | 10 μM DEX vs. VEH | 0.022 ± 0.005 vs. 0.015 ± 0.009 | P = 0.0001 | 1-way ANOVA with Tukey’s multiple comparison test | Fig. 1B |
| FBs | Decay | 1 μM DEX vs. VEH | 0.023 ± 0.004 vs. 0.015 ± 0.009 | P < 0.0001 | 1-way ANOVA with Tukey’s multiple comparison test | Fig. 1B |
| FBs | Decay | DEX 100 µM + RES 5 µM vs. VEH | 0.024 ± 0.006 vs. 0.015 ± 0.009 | P < 0.0001 | 1-way ANOVA with Tukey’s multiple comparison test | Fig. 1B |
| FBs | Decay | DEX 10 µM + RES 5 µM vs. VEH | 0.023 ± 0.004 vs. 0.015 ± 0.009 | P < 0.0001 | 1-way ANOVA with Tukey’s multiple comparison test | Fig. 1B |
| FBs | Decay | DEX 1 µM + RES 5 µM vs. VEH | 0.024 ± 0.005 vs. 0.015 ± 0.009 | P < 0.0001 | 1-way ANOVA with Tukey’s multiple comparison test | Fig. 1B |
| FBs | Decay | DEX 10 µM + RES 25 µM vs. VEH | 0.022 ± 0.003 vs. 0.015 ± 0.009 | P = 0.0021 | 1-way ANOVA with Tukey’s multiple comparison test | Fig. 1B |
| FBs | Decay | 5 μM RES vs VEH | 0.007 ± 0.008 vs. 0.015 ± 0.009 | P = 0.0002 | 1-way ANOVA with Tukey’s multiple comparison test | Fig. 1B |
| FBs | Decay | 25 μM RES vs. VEH | 0.017 ± 0.009 vs. 0.015 ± 0.009 | P = 0.9961 | 1-way ANOVA with Tukey’s multiple comparison test | Fig. 1B |
| CMCs | Trend | 5 μM RES vs. VEH | 0.013 ± 0.007 vs. 0.015 ± 0.006 | P > 0.9999 | 1-way ANOVA with Tukey’s multiple comparison test | Fig. 1A |
| CMCs | Trend | 25 μM RES vs. VEH | 0.009 ± 0.005 vs. 0.015 ± 0.006 | P = 0.4674 | 1-way ANOVA with Tukey’s multiple comparison test | Fig. 1A |
| FBs | Trend | 10 μM DEX vs. DEX 10 µM + RES 25 µM | 0.027 ± 0.007 vs. 0.014 ± 0.005 | P < 0.0001 | 1-way ANOVA with Tukey’s multiple comparison test | Fig. 1B |
| FBs | Trend | 1 μM DEX vs. DEX 1 µM + RES 25 µM | 0.028 ± 0.008 vs. 0.013 ± 0.004 | P < 0.0001 | 1-way ANOVA with Tukey’s multiple comparison test | Fig. 1B |
| FBs | Trend | DEX 10 µM + RES 25 µM vs. VEH | 0.014 ± 0.005 vs. 0.023 ± 0.01 | P = 0.0178 | 1-way ANOVA with Tukey’s multiple comparison test | Fig. 1B |
| FBs | Trend | DEX 1 µM + RES 25 µM vs. VEH | 0.013 ± 0.004 vs. 0.023 ± 0.01 | P = 0.0203 | 1-way ANOVA with Tukey’s multiple comparison test | Fig. 1B |
| FBs | Trend | 5 μM RES vs VEH | 0.024 ± 0.006 vs. 0.023 ± 0.01 | P = 0.9993 | 1-way ANOVA with Tukey’s multiple comparison test | Fig. 1B |
| FBs | Trend | 25 μM RES vs. VEH | 0.012 ± 0.003 vs. 0.023 ± 0.01 | P = 0.0020 | 1-way ANOVA with Tukey’s multiple comparison test | Fig. 1B |

1. **RES prolongs the PER2::LUC half-life via the cAMP-dependent pathway in the fetal CMCs (Fig. 2)**

| **Cell type** | **Parameter** | **Groups** | **Values** | **P value** | **Statistical method** | **Figure** |
| --- | --- | --- | --- | --- | --- | --- |
| CMCs | Half-life | DEX 100 µM vs. VEH | 0.92 ± 0.008 h vs. 1.09 ± 0.05 h | P = 0.8423 | 1-way ANOVA with Tukey’s multiple comparison test | Fig. 2A |
| CMCs | Half-life | DEX 100 µM vs. RES 25 µM | 0.92 ± 0.008 h vs. 2.35 ± 0.494 h | P < 0.0001 | 1-way ANOVA with Tukey’s multiple comparison test | Fig. 2A |
| CMCs | Half-life | DEX 100 µM vs. DEX 100 µM + RES 25 µM | 0.92 ± 0.008 h vs. 2.18 ± 0.308 h | P = 0.0003 | 1-way ANOVA with Tukey’s multiple comparison test | Fig. 2A |
| CMCs | Half-life | RES 25 µM vs. VEH | 2.35 ± 0.494 h vs. 1.09 ± 0.05 h | P = 0.0003 | 1-way ANOVA with Tukey’s multiple comparison test | Fig. 2A |
| CMCs | Half-life | DEX 100 µM + RES 25 µM vs. VEH | 2.18 ± 0.308 h vs. 1.09 ± 0.05 h | P = 0.0010 | 1-way ANOVA with Tukey’s multiple comparison test | Fig. 2A |
| CMCs | K | DEX 100 µM vs. VEH | 0.753 ± 0.006 h vs. 0.636 ± 0.031 | P = 0.0070 | 1-way ANOVA with Tukey’s multiple comparison test | Fig. 2A |
| CMCs | K | DEX 100 µM vs. RES 25 µM | 0.753 ± 0.006 h vs. 0.304 ± 0.058 | P < 0.0001 | 1-way ANOVA with Tukey’s multiple comparison test | Fig. 2A |
| CMCs | K | DEX 100 µM vs. DEX 100 µM + RES 25 µM | 0.753 ± 0.006 h vs. 0.323 ± 0.046 | P < 0.0001 | 1-way ANOVA with Tukey’s multiple comparison test | Fig. 2A |
| CMCs | K | RES 25 µM vs. VEH | 0.304 ± 0.058 h vs. 0.636 ± 0.031 | P < 0.0001 | 1-way ANOVA with Tukey’s multiple comparison test | Fig. 2A |
| CMCs | K | DEX 100 µM + RES 25 µM vs. VEH | 0.323 ± 0.046 h vs. 0.636 ± 0.031 | P = 0.0037 | 1-way ANOVA with Tukey’s multiple comparison test | Fig. 2A |
| FBs | Half-life | DEX 100 µM vs. VEH | 0.864 ± 0.062 h vs. 1.245 ± 0.058 h | P < 0.0001 | 1-way ANOVA with Tukey’s multiple comparison test | Fig. 2B |
| FBs | Half-life | DEX 100 µM vs. RES 25 µM | 0.864 ± 0.062 h vs. 1.981 ± 0.223 h | P < 0.0001 | 1-way ANOVA with Tukey’s multiple comparison test | Fig. 2B |
| FBs | Half-life | DEX 100 µM vs. DEX 100 µM + RES 25 µM | 0.864 ± 0.062 h vs. 0.883 ± 0.026 h | P = 0.9933 | 1-way ANOVA with Tukey’s multiple comparison test | Fig. 2B |
| FBs | Half-life | RES 25 µM vs. VEH | 1.981 ± 0.223 vs. 1.245 ± 0.058 h | P < 0.0001 | 1-way ANOVA with Tukey’s multiple comparison test | Fig. 2B |
| FBs | Half-life | DEX 100 µM + RES 25 µM vs. VEH | 0.883 ± 0.026 vs. 1.245 ± 0.058 h | P = 0.0002 | 1-way ANOVA with Tukey’s multiple comparison test | Fig. 2B |
| FBs | K | DEX 100 µM vs. VEH | 0.805 ± 0.053 h vs. 0.558 ± 0.025 | P < 0.0001 | 1-way ANOVA with Tukey’s multiple comparison test | Fig. 2B |
| FBs | K | DEX 100 µM vs. RES 25 µM | 0.805 ± 0.053 h vs. 0.354 ± 0.042 | P < 0.0001 | 1-way ANOVA with Tukey’s multiple comparison test | Fig. 2B |
| FBs | K | DEX 100 µM vs. DEX 100 µM + RES 25 µM | 0.805 ± 0.053 h vs. 0.786 ± 0.024 | P = 0.8167 | 1-way ANOVA with Tukey’s multiple comparison test | Fig. 2B |
| FBs | K | RES 25 µM vs. VEH | 0.354 ± 0.042 h vs. 0.558 ± 0.025 | P < 0.0001 | 1-way ANOVA with Tukey’s multiple comparison test | Fig. 2B |
| FBs | K | DEX 100 µM + RES 25 µM vs. VEH | 0.786 ± 0.024 h vs. 0.558 ± 0.025 | P < 0.0001 | 1-way ANOVA with Tukey’s multiple comparison test | Fig. 2B |
| CMCs | Half-life | VEH vs. RES 25 μM | 0.88 ± 0.03 h vs. 2.1 ± 0.25 h | P < 0.0001 | 1-way ANOVA with Tukey’s multiple comparison test | Fig. 2C |
| CMCs | Half-life | VEH vs. RES 25 μM + ACi | 0.88 ± 0.03 h vs. 1.43 ± 0.14 h | P < 0.0001 | 1-way ANOVA with Tukey’s multiple comparison test | Fig. 2C |
| CMCs | Half-life | RES 25 μM vs. ACi | 2.1 ± 0.25 h vs. 0.78 ± 0.06 h | P < 0.0001 | 1-way ANOVA with Tukey’s multiple comparison test | Fig. 2C |
| CMCs | Half-life | RES 25 μM vs. RES 25 μM + ACi | 2.1 ± 0.25 h vs. 1.43 ± 0.14 h | P < 0.0001 | 1-way ANOVA with Tukey’s multiple comparison test | Fig. 2C |
| CMCs | Half-life | ACi vs. RES 25 μM + ACi | 0.78 ± 0.06 h vs. 1.43 ± 0.14 h | P < 0.0001 | 1-way ANOVA with Tukey’s multiple comparison test | Fig. 2C |
| CMCs | K | VEH vs. RES 25 μM | 0.79 ± 0.03 vs. 0.34 ± 0.03 | P < 0.0001 | 1-way ANOVA with Tukey’s multiple comparison test | Fig. 2C |
| CMCs | K | VEH vs. ACi | 0.79 ± 0.03 vs. 0.89 ± 0.07 | P = 0.0069 | 1-way ANOVA with Tukey’s multiple comparison test | Fig. 2C |
| CMCs | K | VEH vs. RES 25 μM + ACi | 0.79 ± 0.03 vs. 0.49 ± 0.05 | P < 0.0001 | 1-way ANOVA with Tukey’s multiple comparison test | Fig. 2C |
| CMCs | K | RES 25 μM vs. ACi | 0.34 ± 0.03 vs. 0.89 ± 0.07 | P < 0.0001 | 1-way ANOVA with Tukey’s multiple comparison test | Fig. 2C |
| CMCs | K | RES 25 μM vs. RES 25 μM + ACi | 0.34 ± 0.03 vs. 0.49 ± 0.05 | P = 0.0001 | 1-way ANOVA with Tukey’s multiple comparison test | Fig. 2C |
| CMCs | K | ACi vs. RES 25 μM + ACi | 0.89 ± 0.07 vs. 0.49 ± 0.05 | P < 0.0001 | 1-way ANOVA with Tukey’s multiple comparison test | Fig. 2C |
| FBs | Half-life | VEH vs. RES 25 μM | 0.69 ± 0.05 h vs. 0.85 ± 0.1 h | P = 0.0175 | 1-way ANOVA with Tukey’s multiple comparison test | Fig. 2D |
| FBs | Half-life | VEH vs. RES 25 μM + ACi | 0.69 ± 0.05 h vs. 1.21 ± 0.1 h | P < 0.0001 | 1-way ANOVA with Tukey’s multiple comparison test | Fig. 2D |
| FBs | Half-life | RES 25 μM vs. RES 25 μM + ACi | 0.85 ± 0.1 h vs. 1.21 ± 0.1 h | P < 0.0001 | 1-way ANOVA with Tukey’s multiple comparison test | Fig. 2D |
| FBs | Half-life | ACi vs. RES 25 μM + ACi | 0.74 ± 0.06 h vs. 1.21 ± 0.1 h | P < 0.0001 | 1-way ANOVA with Tukey’s multiple comparison test | Fig. 2D |
| FBs | K | VEH vs. RES 25 μM | 1.01 ± 0.08 vs. 0.83 ± 0.11 | P = 0.0049 | 1-way ANOVA with Tukey’s multiple comparison test | Fig. 2D |
| FBs | K | VEH vs. RES 25 μM + ACi | 1.01 ± 0.08 vs. 0.58 ± 0.05 | P < 0.0001 | 1-way ANOVA with Tukey’s multiple comparison test | Fig. 2D |
| FBs | K | RES 25 μM vs. RES 25 μM + ACi | 0.83 ± 0.11 vs. 0.58 ± 0.05 | P = 0.0001 | 1-way ANOVA with Tukey’s multiple comparison test | Fig. 2D |
| FBs | K | ACi vs. RES 25 μM + ACi | 0.94 ± 0.07 vs. 0.58 ± 0.05 | P < 0.0001 | 1-way ANOVA with Tukey’s multiple comparison test | Fig. 2D |

1. **Treatment with RES and DEX resulted in a circadian but not ATP phase shift in CMCs and FBs (Fig. 3)**

| **Cell type** | **Parameter** | **Time point** | **Groups** | **P value** | **Statistical method** | **Figure** |
| --- | --- | --- | --- | --- | --- | --- |
| CMCs | ATP | 0 hours | Dexamethasone 100μM vs. Resveratrol 25μM | P = 0.0386 | 2-way ANOVA with Tukey’s multiple comparison test | Fig. 3F |
| CMCs | ATP | 0 hours | Dexamethasone 100μM vs. Res+Dex 100μM | P = 0.948 | 2-way ANOVA with Tukey’s multiple comparison test | Fig. 3F |
| CMCs | ATP | 0 hours | Dexamethasone 100μM vs. VEH | P = 0.001 | 2-way ANOVA with Tukey’s multiple comparison test | Fig. 3F |
| CMCs | ATP | 0 hours | Resveratrol 25μM vs. Res+Dex 100μM | P = 0.033 | 2-way ANOVA with Tukey’s multiple comparison test | Fig. 3F |
| CMCs | ATP | 0 hours | Resveratrol 25μM vs. VEH | P = 0.016 | 2-way ANOVA with Tukey’s multiple comparison test | Fig. 3F |
| CMCs | ATP | 0 hours | Res+Dex 100μM vs. VEH | P = 0.0025 | 2-way ANOVA with Tukey’s multiple comparison test | Fig. 3F |
| CMCs | ATP | 6 hours | Dexamethasone 100μM vs. Resveratrol 25μM | P = 0.0564 | 2-way ANOVA with Tukey’s multiple comparison test | Fig. 3F |
| CMCs | ATP | 6 hours | Dexamethasone 100μM vs. Res+Dex 100μM | P = 0.0485 | 2-way ANOVA with Tukey’s multiple comparison test | Fig. 3F |
| CMCs | ATP | 6 hours | Dexamethasone 100μM vs. VEH | P = 0.0178 | 2-way ANOVA with Tukey’s multiple comparison test | Fig. 3F |
| CMCs | ATP | 6 hours | Resveratrol 25μM vs. Res+Dex 100μM | P = 0.8321 | 2-way ANOVA with Tukey’s multiple comparison test | Fig. 3F |
| CMCs | ATP | 6 hours | Resveratrol 25μM vs. VEH | P = 0.0247 | 2-way ANOVA with Tukey’s multiple comparison test | Fig. 3F |
| CMCs | ATP | 6 hours | Res+Dex 100μM vs. VEH | P = 0.0259 | 2-way ANOVA with Tukey’s multiple comparison test | Fig. 3F |
| CMCs | ATP | 12 hours | Dexamethasone 100μM vs. Resveratrol 25μM | P = 0.0047 | 2-way ANOVA with Tukey’s multiple comparison test | Fig. 3F |
| CMCs | ATP | 12 hours | Dexamethasone 100μM vs. Res+Dex 100μM | P = 0.1803 | 2-way ANOVA with Tukey’s multiple comparison test | Fig. 3F |
| CMCs | ATP | 12 hours | Dexamethasone 100μM vs. VEH | P = 0.0099 | 2-way ANOVA with Tukey’s multiple comparison test | Fig. 3F |
| CMCs | ATP | 12 hours | Resveratrol 25μM vs. Res+Dex 100μM | P = 0.1416 | 2-way ANOVA with Tukey’s multiple comparison test | Fig. 3F |
| CMCs | ATP | 12 hours | Resveratrol 25μM vs. VEH | P = 0.0168 | 2-way ANOVA with Tukey’s multiple comparison test | Fig. 3F |
| CMCs | ATP | 12 hours | Res+Dex 100μM vs. VEH | P = 0.0049 | 2-way ANOVA with Tukey’s multiple comparison test | Fig. 3F |
| CMCs | ATP | 18 hours | Dexamethasone 100μM vs. Resveratrol 25μM | P = 0.01 | 2-way ANOVA with Tukey’s multiple comparison test | Fig. 3F |
| CMCs | ATP | 18 hours | Dexamethasone 100μM vs. Res+Dex 100μM | P = 0.2032 | 2-way ANOVA with Tukey’s multiple comparison test | Fig. 3F |
| CMCs | ATP | 18 hours | Dexamethasone 100μM vs. VEH | P = 0.0002 | 2-way ANOVA with Tukey’s multiple comparison test | Fig. 3F |
| CMCs | ATP | 18 hours | Resveratrol 25μM vs. Res+Dex 100μM | P = 0.0408 | 2-way ANOVA with Tukey’s multiple comparison test | Fig. 3F |
| CMCs | ATP | 18 hours | Resveratrol 25μM vs. VEH | P = 0.0009 | 2-way ANOVA with Tukey’s multiple comparison test | Fig. 3F |
| CMCs | ATP | 18 hours | Res+Dex 100μM vs. VEH | P = 0.0013 | 2-way ANOVA with Tukey’s multiple comparison test | Fig. 3F |
| CMCs | ATP | 24 hours | Dexamethasone 100μM vs. Resveratrol 25μM | P = 0.2117 | 2-way ANOVA with Tukey’s multiple comparison test | Fig. 3F |
| CMCs | ATP | 24 hours | Dexamethasone 100μM vs. Res+Dex 100μM | P = 0.4658 | 2-way ANOVA with Tukey’s multiple comparison test | Fig. 3F |
| CMCs | ATP | 24 hours | Dexamethasone 100μM vs. VEH | P = 0.0294 | 2-way ANOVA with Tukey’s multiple comparison test | Fig. 3F |
| CMCs | ATP | 24 hours | Resveratrol 25μM vs. Res+Dex 100μM | P = 0.0993 | 2-way ANOVA with Tukey’s multiple comparison test | Fig. 3F |
| CMCs | ATP | 24 hours | Resveratrol 25μM vs. VEH | P = 0.0524 | 2-way ANOVA with Tukey’s multiple comparison test | Fig. 3F |
| CMCs | ATP | 24 hours | Res+Dex 100μM vs. VEH | P = 0.019 | 2-way ANOVA with Tukey’s multiple comparison test | Fig. 3F |

1. **The rhythm of mitochondrial network morphology is synchronized in all treatment groups in FBs (Fig. 4)**

| **Cell type** | **Parameter** | **Time point** | **Groups** | **P value** | **Statistical method** | **Figure** |
| --- | --- | --- | --- | --- | --- | --- |
| FBs | Total Branch Length/mito | 0 hours | Dexamethasone 100μM vs. Resveratrol 25μM | P = 0.0009 | 2-way mixed ANOVA with Tukey’s multiple comparison test | Fig. 4B |
| FBs | Total Branch Length/mito | 0 hours | Dexamethasone 100μM vs. Res+Dex 100μM | P < 0.0001 | 2-way mixed ANOVA with Tukey’s multiple comparison test | Fig. 4B |
| FBs | Total Branch Length/mito | 0 hours | Dexamethasone 100μM vs. VEH | P < 0.0001 | 2-way mixed ANOVA with Tukey’s multiple comparison test | Fig. 4B |
| FBs | Total Branch Length/mito | 0 hours | Resveratrol 25μM vs. Res+Dex 100μM | P = 0.6413 | 2-way mixed ANOVA with Tukey’s multiple comparison test | Fig. 4B |
| FBs | Total Branch Length/mito | 0 hours | Resveratrol 25μM vs. VEH | P < 0.0001 | 2-way mixed ANOVA with Tukey’s multiple comparison test | Fig. 4B |
| FBs | Total Branch Length/mito | 0 hours | Res+Dex 100μM vs. VEH | P = 0.0014 | 2-way mixed ANOVA with Tukey’s multiple comparison test | Fig. 4B |
| FBs | Total Branch Length/mito | 6 hours | Dexamethasone 100μM vs. Resveratrol 25μM | P = 0.0033 | 2-way mixed ANOVA with Tukey’s multiple comparison test | Fig. 4B |
| FBs | Total Branch Length/mito | 6 hours | Dexamethasone 100μM vs. Res+Dex 100μM | P = 0.0086 | 2-way mixed ANOVA with Tukey’s multiple comparison test | Fig. 4B |
| FBs | Total Branch Length/mito | 6 hours | Dexamethasone 100μM vs. VEH | P = 0.3301 | 2-way mixed ANOVA with Tukey’s multiple comparison test | Fig. 4B |
| FBs | Total Branch Length/mito | 6 hours | Resveratrol 25μM vs. Res+Dex 100μM | P = 0.9943 | 2-way mixed ANOVA with Tukey’s multiple comparison test | Fig. 4B |
| FBs | Total Branch Length/mito | 6 hours | Resveratrol 25μM vs. VEH | P = 0.374 | 2-way mixed ANOVA with Tukey’s multiple comparison test | Fig. 4B |
| FBs | Total Branch Length/mito | 6 hours | Res+Dex 100μM vs. VEH | P = 0.527 | 2-way mixed ANOVA with Tukey’s multiple comparison test | Fig. 4B |
| FBs | Total Branch Length/mito | 12 hours | Dexamethasone 100μM vs. Resveratrol 25μM | P < 0.0001 | 2-way mixed ANOVA with Tukey’s multiple comparison test | Fig. 4B |
| FBs | Total Branch Length/mito | 12 hours | Dexamethasone 100μM vs. Res+Dex 100μM | P < 0.0001 | 2-way mixed ANOVA with Tukey’s multiple comparison test | Fig. 4B |
| FBs | Total Branch Length/mito | 12 hours | Dexamethasone 100μM vs. VEH | P < 0.0001 | 2-way mixed ANOVA with Tukey’s multiple comparison test | Fig. 4B |
| FBs | Total Branch Length/mito | 12 hours | Resveratrol 25μM vs. Res+Dex 100μM | P = 0.9977 | 2-way mixed ANOVA with Tukey’s multiple comparison test | Fig. 4B |
| FBs | Total Branch Length/mito | 12 hours | Resveratrol 25μM vs. VEH | P = 0.0178 | 2-way mixed ANOVA with Tukey’s multiple comparison test | Fig. 4B |
| FBs | Total Branch Length/mito | 12 hours | Res+Dex 100μM vs. VEH | P = 0.0649 | 2-way mixed ANOVA with Tukey’s multiple comparison test | Fig. 4B |
| FBs | Total Branch Length/mito | 18 hours | Dexamethasone 100μM vs. Resveratrol 25μM | P = 0.071 | 2-way mixed ANOVA with Tukey’s multiple comparison test | Fig. 4B |
| FBs | Total Branch Length/mito | 18 hours | Dexamethasone 100μM vs. Res+Dex 100μM | P = 0.0775 | 2-way mixed ANOVA with Tukey’s multiple comparison test | Fig. 4B |
| FBs | Total Branch Length/mito | 18 hours | Dexamethasone 100μM vs. VEH | P < 0.0001 | 2-way mixed ANOVA with Tukey’s multiple comparison test | Fig. 4B |
| FBs | Total Branch Length/mito | 18 hours | Resveratrol 25μM vs. Res+Dex 100μM | P = 0.9833 | 2-way mixed ANOVA with Tukey’s multiple comparison test | Fig. 4B |
| FBs | Total Branch Length/mito | 18 hours | Resveratrol 25μM vs. VEH | P = 0.0139 | 2-way mixed ANOVA with Tukey’s multiple comparison test | Fig. 4B |
| FBs | Total Branch Length/mito | 18 hours | Res+Dex 100μM vs. VEH | P = 0.0713 | 2-way mixed ANOVA with Tukey’s multiple comparison test | Fig. 4B |
| FBs | Total Branch Length/mito | 24 hours | Dexamethasone 100μM vs. Resveratrol 25μM | P = 0.9456 | 2-way mixed ANOVA with Tukey’s multiple comparison test | Fig. 4B |
| FBs | Total Branch Length/mito | 24 hours | Dexamethasone 100μM vs. Res+Dex 100μM | P = 0.9978 | 2-way mixed ANOVA with Tukey’s multiple comparison test | Fig. 4B |
| FBs | Total Branch Length/mito | 24 hours | Dexamethasone 100μM vs. VEH | P = 0.0006 | 2-way mixed ANOVA with Tukey’s multiple comparison test | Fig. 4B |
| FBs | Total Branch Length/mito | 24 hours | Resveratrol 25μM vs. Res+Dex 100μM | P = 0.8928 | 2-way mixed ANOVA with Tukey’s multiple comparison test | Fig. 4B |
| FBs | Total Branch Length/mito | 24 hours | Resveratrol 25μM vs. VEH | P = 0.0019 | 2-way mixed ANOVA with Tukey’s multiple comparison test | Fig. 4B |
| FBs | Total Branch Length/mito | 24 hours | Res+Dex 100μM vs. VEH | P = 0.0006 | 2-way mixed ANOVA with Tukey’s multiple comparison test | Fig. 4B |
| FBs | Mean Form Factor | 0 hours | Dexamethasone 100μM vs. Resveratrol 25μM | P < 0.0001 | 2-way mixed ANOVA with Tukey’s multiple comparison test | Fig. 4B |
| FBs | Mean Form Factor | 0 hours | Dexamethasone 100μM vs. Res+Dex 100μM | P < 0.0001 | 2-way mixed ANOVA with Tukey’s multiple comparison test | Fig. 4B |
| FBs | Mean Form Factor | 0 hours | Dexamethasone 100μM vs. VEH | P = 0.6031 | 2-way mixed ANOVA with Tukey’s multiple comparison test | Fig. 4B |
| FBs | Mean Form Factor | 0 hours | Resveratrol 25μM vs. Res+Dex 100μM | P = 0.855 | 2-way mixed ANOVA with Tukey’s multiple comparison test | Fig. 4B |
| FBs | Mean Form Factor | 0 hours | Resveratrol 25μM vs. VEH | P = 0.0002 | 2-way mixed ANOVA with Tukey’s multiple comparison test | Fig. 4B |
| FBs | Mean Form Factor | 0 hours | Res+Dex 100μM vs. VEH | P = 0.0016 | 2-way mixed ANOVA with Tukey’s multiple comparison test | Fig. 4B |
| FBs | Mean Form Factor | 6 hours | Dexamethasone 100μM vs. Resveratrol 25μM | P = 0.7483 | 2-way mixed ANOVA with Tukey’s multiple comparison test | Fig. 4B |
| FBs | Mean Form Factor | 6 hours | Dexamethasone 100μM vs. Res+Dex 100μM | P = 0.0161 | 2-way mixed ANOVA with Tukey’s multiple comparison test | Fig. 4B |
| FBs | Mean Form Factor | 6 hours | Dexamethasone 100μM vs. VEH | P = 0.0769 | 2-way mixed ANOVA with Tukey’s multiple comparison test | Fig. 4B |
| FBs | Mean Form Factor | 6 hours | Resveratrol 25μM vs. Res+Dex 100μM | P = 0.3544 | 2-way mixed ANOVA with Tukey’s multiple comparison test | Fig. 4B |
| FBs | Mean Form Factor | 6 hours | Resveratrol 25μM vs. VEH | P = 0.0143 | 2-way mixed ANOVA with Tukey’s multiple comparison test | Fig. 4B |
| FBs | Mean Form Factor | 6 hours | Res+Dex 100μM vs. VEH | P < 0.0001 | 2-way mixed ANOVA with Tukey’s multiple comparison test | Fig. 4B |
| FBs | Mean Form Factor | 12 hours | Dexamethasone 100μM vs. Resveratrol 25μM | P = 0.4398 | 2-way mixed ANOVA with Tukey’s multiple comparison test | Fig. 4B |
| FBs | Mean Form Factor | 12 hours | Dexamethasone 100μM vs. Res+Dex 100μM | P = 0.0161 | 2-way mixed ANOVA with Tukey’s multiple comparison test | Fig. 4B |
| FBs | Mean Form Factor | 12 hours | Dexamethasone 100μM vs. VEH | P > 0.9999 | 2-way mixed ANOVA with Tukey’s multiple comparison test | Fig. 4B |
| FBs | Mean Form Factor | 12 hours | Resveratrol 25μM vs. Res+Dex 100μM | P = 0.2061 | 2-way mixed ANOVA with Tukey’s multiple comparison test | Fig. 4B |
| FBs | Mean Form Factor | 12 hours | Resveratrol 25μM vs. VEH | P = 0.4659 | 2-way mixed ANOVA with Tukey’s multiple comparison test | Fig. 4B |
| FBs | Mean Form Factor | 12 hours | Res+Dex 100μM vs. VEH | P = 0.0211 | 2-way mixed ANOVA with Tukey’s multiple comparison test | Fig. 4B |
| FBs | Mean Form Factor | 18 hours | Dexamethasone 100μM vs. Resveratrol 25μM | P = 0.2766 | 2-way mixed ANOVA with Tukey’s multiple comparison test | Fig. 4B |
| FBs | Mean Form Factor | 18 hours | Dexamethasone 100μM vs. Res+Dex 100μM | P = 0.9965 | 2-way mixed ANOVA with Tukey’s multiple comparison test | Fig. 4B |
| FBs | Mean Form Factor | 18 hours | Dexamethasone 100μM vs. VEH | P < 0.0001 | 2-way mixed ANOVA with Tukey’s multiple comparison test | Fig. 4B |
| FBs | Mean Form Factor | 18 hours | Resveratrol 25μM vs. Res+Dex 100μM | P = 0.5963 | 2-way mixed ANOVA with Tukey’s multiple comparison test | Fig. 4B |
| FBs | Mean Form Factor | 18 hours | Resveratrol 25μM vs. VEH | P = 0.0006 | 2-way mixed ANOVA with Tukey’s multiple comparison test | Fig. 4B |
| FBs | Mean Form Factor | 18 hours | Res+Dex 100μM vs. VEH | P < 0.0001 | 2-way mixed ANOVA with Tukey’s multiple comparison test | Fig. 4B |
| FBs | Mean Form Factor | 24 hours | Dexamethasone 100μM vs. Resveratrol 25μM | P = 0.0065 | 2-way mixed ANOVA with Tukey’s multiple comparison test | Fig. 4B |
| FBs | Mean Form Factor | 24 hours | Dexamethasone 100μM vs. Res+Dex 100μM | P = 0.453 | 2-way mixed ANOVA with Tukey’s multiple comparison test | Fig. 4B |
| FBs | Mean Form Factor | 24 hours | Dexamethasone 100μM vs. VEH | P < 0.0001 | 2-way mixed ANOVA with Tukey’s multiple comparison test | Fig. 4B |
| FBs | Mean Form Factor | 24 hours | Resveratrol 25μM vs. Res+Dex 100μM | P = 0.396 | 2-way mixed ANOVA with Tukey’s multiple comparison test | Fig. 4B |
| FBs | Mean Form Factor | 24 hours | Resveratrol 25μM vs. VEH | P = 0.0017 | 2-way mixed ANOVA with Tukey’s multiple comparison test | Fig. 4B |
| FBs | Mean Form Factor | 24 hours | Res+Dex 100μM vs. VEH | P < 0.0001 | 2-way mixed ANOVA with Tukey’s multiple comparison test | Fig. 4B |
| FBs | Branch Junctions/mito | 0 hours | Dexamethasone 100μM vs. Resveratrol 25μM | P = 0.1847 | 2-way mixed ANOVA with Tukey’s multiple comparison test | Fig. 4B |
| FBs | Branch Junctions/mito | 0 hours | Dexamethasone 100μM vs. Res+Dex 100μM | P = 0.0543 | 2-way mixed ANOVA with Tukey’s multiple comparison test | Fig. 4B |
| FBs | Branch Junctions/mito | 0 hours | Dexamethasone 100μM vs. VEH | P < 0.0001 | 2-way mixed ANOVA with Tukey’s multiple comparison test | Fig. 4B |
| FBs | Branch Junctions/mito | 0 hours | Resveratrol 25μM vs. Res+Dex 100μM | P = 0.8825 | 2-way mixed ANOVA with Tukey’s multiple comparison test | Fig. 4B |
| FBs | Branch Junctions/mito | 0 hours | Resveratrol 25μM vs. VEH | P = 0.0043 | 2-way mixed ANOVA with Tukey’s multiple comparison test | Fig. 4B |
| FBs | Branch Junctions/mito | 0 hours | Res+Dex 100μM vs. VEH | P = 0.0193 | 2-way mixed ANOVA with Tukey’s multiple comparison test | Fig. 4B |
| FBs | Branch Junctions/mito | 6 hours | Dexamethasone 100μM vs. Resveratrol 25μM | P = 0.1466 | 2-way mixed ANOVA with Tukey’s multiple comparison test | Fig. 4B |
| FBs | Branch Junctions/mito | 6 hours | Dexamethasone 100μM vs. Res+Dex 100μM | P = 0.3752 | 2-way mixed ANOVA with Tukey’s multiple comparison test | Fig. 4B |
| FBs | Branch Junctions/mito | 6 hours | Dexamethasone 100μM vs. VEH | P = 0.3378 | 2-way mixed ANOVA with Tukey’s multiple comparison test | Fig. 4B |
| FBs | Branch Junctions/mito | 6 hours | Resveratrol 25μM vs. Res+Dex 100μM | P = 0.0008 | 2-way mixed ANOVA with Tukey’s multiple comparison test | Fig. 4B |
| FBs | Branch Junctions/mito | 6 hours | Resveratrol 25μM vs. VEH | P = 0.9627 | 2-way mixed ANOVA with Tukey’s multiple comparison test | Fig. 4B |
| FBs | Branch Junctions/mito | 6 hours | Res+Dex 100μM vs. VEH | P = 0.0043 | 2-way mixed ANOVA with Tukey’s multiple comparison test | Fig. 4B |
| FBs | Branch Junctions/mito | 12 hours | Dexamethasone 100μM vs. Resveratrol 25μM | P < 0.0001 | 2-way mixed ANOVA with Tukey’s multiple comparison test | Fig. 4B |
| FBs | Branch Junctions/mito | 12 hours | Dexamethasone 100μM vs. Res+Dex 100μM | P < 0.0001 | 2-way mixed ANOVA with Tukey’s multiple comparison test | Fig. 4B |
| FBs | Branch Junctions/mito | 12 hours | Dexamethasone 100μM vs. VEH | P < 0.0001 | 2-way mixed ANOVA with Tukey’s multiple comparison test | Fig. 4B |
| FBs | Branch Junctions/mito | 12 hours | Resveratrol 25μM vs. Res+Dex 100μM | P > 0.9999 | 2-way mixed ANOVA with Tukey’s multiple comparison test | Fig. 4B |
| FBs | Branch Junctions/mito | 12 hours | Resveratrol 25μM vs. VEH | P = 0.0211 | 2-way mixed ANOVA with Tukey’s multiple comparison test | Fig. 4B |
| FBs | Branch Junctions/mito | 12 hours | Res+Dex 100μM vs. VEH | P = 0.0218 | 2-way mixed ANOVA with Tukey’s multiple comparison test | Fig. 4B |
| FBs | Branch Junctions/mito | 18 hours | Dexamethasone 100μM vs. Resveratrol 25μM | P = 0.0001 | 2-way mixed ANOVA with Tukey’s multiple comparison test | Fig. 4B |
| FBs | Branch Junctions/mito | 18 hours | Dexamethasone 100μM vs. Res+Dex 100μM | P = 0.7829 | 2-way mixed ANOVA with Tukey’s multiple comparison test | Fig. 4B |
| FBs | Branch Junctions/mito | 18 hours | Dexamethasone 100μM vs. VEH | P = 0.2361 | 2-way mixed ANOVA with Tukey’s multiple comparison test | Fig. 4B |
| FBs | Branch Junctions/mito | 18 hours | Resveratrol 25μM vs. Res+Dex 100μM | P = 0.0357 | 2-way mixed ANOVA with Tukey’s multiple comparison test | Fig. 4B |
| FBs | Branch Junctions/mito | 18 hours | Resveratrol 25μM vs. VEH | P = 0.0006 | 2-way mixed ANOVA with Tukey’s multiple comparison test | Fig. 4B |
| FBs | Branch Junctions/mito | 18 hours | Res+Dex 100μM vs. VEH | P = 0.9496 | 2-way mixed ANOVA with Tukey’s multiple comparison test | Fig. 4B |
| FBs | Branch Junctions/mito | 24 hours | Dexamethasone 100μM vs. Resveratrol 25μM | P = 0.7779 | 2-way mixed ANOVA with Tukey’s multiple comparison test | Fig. 4B |
| FBs | Branch Junctions/mito | 24 hours | Dexamethasone 100μM vs. Res+Dex 100μM | P = 0.0327 | 2-way mixed ANOVA with Tukey’s multiple comparison test | Fig. 4B |
| FBs | Branch Junctions/mito | 24 hours | Dexamethasone 100μM vs. VEH | P = 0.9998 | 2-way mixed ANOVA with Tukey’s multiple comparison test | Fig. 4B |
| FBs | Branch Junctions/mito | 24 hours | Resveratrol 25μM vs. Res+Dex 100μM | P = 0.1472 | 2-way mixed ANOVA with Tukey’s multiple comparison test | Fig. 4B |
| FBs | Branch Junctions/mito | 24 hours | Resveratrol 25μM vs. VEH | P = 0.8189 | 2-way mixed ANOVA with Tukey’s multiple comparison test | Fig. 4B |
| FBs | Branch Junctions/mito | 24 hours | Res+Dex 100μM vs. VEH | P = 0.0379 | 2-way mixed ANOVA with Tukey’s multiple comparison test | Fig. 4B |

1. **Mitochondrial membrane potential (MMP) and reactive oxygen species (ROS) displayed synchronized rhythm in fetal heart FBs (Fig. 5)**

| **Cell type** | **Parameter** | **Time point** | **Groups** | **P value** | **Statistical method** | **Figure** |
| --- | --- | --- | --- | --- | --- | --- |
| FBs | MMP | 0 hours | Dexamethasone 100μM vs. VEH | P = 0.9915 | 2-way mixed ANOVA with Tukey’s multiple comparison test | Fig. 5C |
| FBs | MMP | 6 hours | Dexamethasone 100μM vs. VEH | P = 0.8919 | 2-way mixed ANOVA with Tukey’s multiple comparison test | Fig. 5C |
| FBs | MMP | 12 hours | Dexamethasone 100μM vs. VEH | P < 0.0001 | 2-way mixed ANOVA with Tukey’s multiple comparison test | Fig. 5C |
| FBs | MMP | 18 hours | Dexamethasone 100μM vs. VEH | P = 0.0011 | 2-way mixed ANOVA with Tukey’s multiple comparison test | Fig. 5C |
| FBs | MMP | 24 hours | Dexamethasone 100μM vs. VEH | P = 0.9997 | 2-way mixed ANOVA with Tukey’s multiple comparison test | Fig. 5C |
| FBs | ROS | 0 hours | Dexamethasone 100μM vs. VEH | P = 0.7173 | 2-way mixed ANOVA with Tukey’s multiple comparison test | Fig. 5C |
| FBs | ROS | 6 hours | Dexamethasone 100μM vs. VEH | P = 0.046 | 2-way mixed ANOVA with Tukey’s multiple comparison test | Fig. 5C |
| FBs | ROS | 12 hours | Dexamethasone 100μM vs. VEH | P = 0.015 | 2-way mixed ANOVA with Tukey’s multiple comparison test | Fig. 5C |
| FBs | ROS | 18 hours | Dexamethasone 100μM vs. VEH | P < 0.0001 | 2-way mixed ANOVA with Tukey’s multiple comparison test | Fig. 5C |
| FBs | ROS | 24 hours | Dexamethasone 100μM vs. VEH | P = 0.0022 | 2-way mixed ANOVA with Tukey’s multiple comparison test | Fig. 5C |

1. **Main effects of ANOVA analysis**

| **Cell type** | **Parameter** | **P value** | **Statistical method** | **Figure** |
| --- | --- | --- | --- | --- |
| CMCs | Period | P < 0.0001 | 1-way ANOVA with Tukey’s multiple comparison test | Fig. 1A |
| CMCs | Amplitude | P < 0.0001 | 1-way ANOVA with Tukey’s multiple comparison test | Fig. 1A |
| CMCs | Decay | P < 0.0001 | 1-way ANOVA with Tukey’s multiple comparison test | Fig. 1A |
| CMCs | Trend | P < 0.0001 | 1-way ANOVA with Tukey’s multiple comparison test | Fig. 1A |
| FBs | Period | P < 0.0001 | 1-way ANOVA with Tukey’s multiple comparison test | Fig. 1B |
| FBs | Amplitude | P < 0.0001 | 1-way ANOVA with Tukey’s multiple comparison test | Fig. 1B |
| FBs | Decay | P < 0.0001 | 1-way ANOVA with Tukey’s multiple comparison test | Fig. 1B |
| FBs | Trend | P < 0.0001 | 1-way ANOVA with Tukey’s multiple comparison test | Fig. 1B |
| CMCs | Decay | P < 0.0001 | 1-way ANOVA with Tukey’s multiple comparison test | Fig. 2A |
| CMCs | K | P < 0.0001 | 1-way ANOVA with Tukey’s multiple comparison test | Fig. 2A |
| FBs | Decay | P < 0.0001 | 1-way ANOVA with Tukey’s multiple comparison test | Fig. 2B |
| FBs | K | P < 0.0001 | 1-way ANOVA with Tukey’s multiple comparison test | Fig. 2B |
| CMCs | Decay | P < 0.0001 | 1-way ANOVA with Tukey’s multiple comparison test | Fig. 2C |
| CMCs | K | P < 0.0001 | 1-way ANOVA with Tukey’s multiple comparison test | Fig. 2C |
| FBs | Decay | P < 0.0001 | 1-way ANOVA with Tukey’s multiple comparison test | Fig. 2D |
| FBs | K | P < 0.0001 | 1-way ANOVA with Tukey’s multiple comparison test | Fig. 2D |
| CMCs | CT9-13 | P < 0.0001 | 1-way ANOVA with Dunnett’s multiple comparison test | Fig. 3C |
| CMCs | CT14-18 | P = 0.0011 | 1-way ANOVA with Dunnett’s multiple comparison test | Fig. 3C |
| FBs | CT9-13 | P < 0.0001 | 1-way ANOVA with Dunnett’s multiple comparison test | Fig. 3D |
| FBs | CT14-18 | P = 0.0018 | 1-way ANOVA with Dunnett’s multiple comparison test | Fig. 3D |
| CMCs | Resazurin (treatment) | P = 0.0016 | 2-way ANOVA with Tukey’s multiple comparison test | Fig. 3E |
|  | Resazurin (time) | P < 0.0001 | 2-way ANOVA with Tukey’s multiple comparison test | Fig. 3E |
|  | Resazurin (Interaction) | P < 0.0001 | 2-way ANOVA with Tukey’s multiple comparison test | Fig. 3E |
| CMCs | ATP (treatment) | P < 0.0001 | 2-way ANOVA with Tukey’s multiple comparison test | Fig. 3F |
|  | ATP (time) | P < 0.0001 | 2-way ANOVA with Tukey’s multiple comparison test | Fig. 3F |
|  | ATP (Interaction) | P = 0.0004 | 2-way ANOVA with Tukey’s multiple comparison test | Fig. 3F |
| FBs | Resazurin (treatment) | P = 0.0879 | 2-way ANOVA with Tukey’s multiple comparison test | Fig. 3G |
|  | Resazurin (time) | P < 0.0001 | 2-way ANOVA with Tukey’s multiple comparison test | Fig. 3G |
|  | Resazurin (Interaction) | P = 0.7671 | 2-way ANOVA with Tukey’s multiple comparison test | Fig. 3G |
| FBs | ATP (treatment) | P = 0.0172 | 2-way ANOVA with Tukey’s multiple comparison test | Fig. 3H |
|  | ATP (time) | P < 0.0001 | 2-way ANOVA with Tukey’s multiple comparison test | Fig. 3H |
|  | ATP (Interaction) | P = 0.0003 | 2-way ANOVA with Tukey’s multiple comparison test | Fig. 3H |
| CMCs | Acrophase (PER2/ATP phase) | P < 0.0001 | 2-way ANOVA with Tukey’s multiple comparison test | Fig. 3I |
|  | Acrophase (treatment) | P = 0.6484 | 2-way ANOVA with Tukey’s multiple comparison test | Fig. 3I |
|  | Acrophase (interaction) | P = 0.7094 | 2-way ANOVA with Tukey’s multiple comparison test | Fig. 3I |
| FBs | Acrophase (PER2/ATP phase) | P < 0.0001 | 2-way ANOVA with Tukey’s multiple comparison test | Fig. 3J |
|  | Acrophase (treatment) | P < 0.0001 | 2-way ANOVA with Tukey’s multiple comparison test | Fig. 3J |
|  | Acrophase (interaction) | P < 0.0001 | 2-way ANOVA with Tukey’s multiple comparison test | Fig. 3J |
| FBs | Total branch length/mito (treatment) | P < 0.0001 | Mixed effect analysis with Tukey’s multiple comparison test | Fig. 4B |
|  | Total branch length/mito (time) | P < 0.0001 | Mixed effect analysis with Tukey’s multiple comparison test | Fig. 4B |
|  | Total branch length/mito (interaction) | P < 0.0001 | Mixed effect analysis with Tukey’s multiple comparison test | Fig. 4B |
| FBs | Branch junctions/mito (treatment) | P < 0.0001 | Mixed effect analysis with Tukey’s multiple comparison test | Fig. 4B |
|  | Branch junctions/mito (time) | P < 0.0001 | Mixed effect analysis with Tukey’s multiple comparison test | Fig. 4B |
|  | Branch junctions/mito (interaction) | P < 0.0001 | Mixed effect analysis with Tukey’s multiple comparison test | Fig. 4B |
| FBs | Mean form factor (treatment) | P < 0.0001 | Mixed effect analysis with Tukey’s multiple comparison test | Fig. 4B |
|  | Mean form factor (time) | P < 0.0001 | Mixed effect analysis with Tukey’s multiple comparison test | Fig. 4B |
|  | Mean form factor (interaction) | P < 0.0001 | Mixed effect analysis with Tukey’s multiple comparison test | Fig. 4B |
| FBs | Mitotracker Red (treatment) | P = 0.8105 | Mixed effect analysis with Sidak’s multiple comparison test | Fig. 5C |
|  | Mitotracker Red (time) | P < 0.0001 | Mixed effect analysis with Sidak’s multiple comparison test | Fig. 5C |
|  | Mitotracker Red (interaction) | P < 0.0001 | Mixed effect analysis with Sidak’s multiple comparison test | Fig. 5C |
| FBs | CellROX (treatment) | P = 0.0004 | Mixed effect analysis with Sidak’s multiple comparison test | Fig. 5C |
|  | CellROX (time) | P < 0.0001 | Mixed effect analysis with Sidak’s multiple comparison test | Fig. 5C |
|  | CellROX (interaction) | P < 0.0001 | Mixed effect analysis with Sidak’s multiple comparison test | Fig. 5C |

1. **Acrophase parameters of the rhythm of mitochondrial function**

| **Cell type** | **Experiment** | **Group** | **Comparison of Fits (P value)** | **Goodness of Fit (R squared)** | **Figure** |
| --- | --- | --- | --- | --- | --- |
| CMCs | Resazurin | Dexamethasone 100μM | P = 0.8693 | 0.02307 | Fig. 3E |
| CMCs | Resazurin | Resveratrol 25μM | P = 0.7842 | 0.0397 | Fig. 3E |
| CMCs | Resazurin | Res 25μM +Dex 100μM | P = 0.1245 | 0.2934 | Fig. 3E |
| CMCs | Resazurin | VEH | P = 0.6495 | 0.06941 | Fig. 3E |
| CMCs | ATP | Dexamethasone 100μM | P = 0.0016 | 0.6581 | Fig. 3F |
| CMCs | ATP | Resveratrol 25μM | P = 0.003 | 0.6212 | Fig. 3F |
| CMCs | ATP | Res 25μM +Dex 100μM | P = 0.0045 | 0.5936 | Fig. 3F |
| CMCs | ATP | VEH | P = 0.0003 | 0.7468 | Fig. 3F |
| FBs | Resazurin | Dexamethasone 100μM | P = 0.0275 | 0.3449 | Fig. 3G |
| FBs | Resazurin | Resveratrol 25μM | P = 0.0093 | 0.4233 | Fig. 3G |
| FBs | Resazurin | Res 25μM +Dex 100μM | P = 0.0147 | 0.3911 | Fig. 3G |
| FBs | Resazurin | VEH | P = 0.1984 | 0.1733 | Fig. 3G |
| FBs | ATP | Dexamethasone 100μM | P = 0.0075 | 0.558 | Fig. 3H |
| FBs | ATP | Resveratrol 25μM | P = 0.0477 | 0.3977 | Fig. 3H |
| FBs | ATP | Res 25μM +Dex 100μM | P = 0.0074 | 0.5587 | Fig. 3H |
| FBs | ATP | VEH | P = 0.0434 | 0.4072 | Fig. 3H |
| FBs | Total Branch Length/mito | Dexamethasone 100μM | P < 0.0001 | 0.2463 | Fig. 4B |
| FBs | Total Branch Length/mito | Resveratrol 25μM | P < 0.0001 | 0.1437 | Fig. 4B |
| FBs | Total Branch Length/mito | Res 25μM +Dex 100μM | P < 0.0001 | 0.1084 | Fig. 4B |
| FBs | Total Branch Length/mito | VEH | P < 0.0001 | 0.1821 | Fig. 4B |
| FBs | Mean Form Factor | Dexamethasone 100μM | P < 0.0001 | 0.1582 | Fig. 4B |
| FBs | Mean Form Factor | Resveratrol 25μM | P < 0.0001 | 0.1306 | Fig. 4B |
| FBs | Mean Form Factor | Res 25μM +Dex 100μM | P < 0.0001 | 0.1689 | Fig. 4B |
| FBs | Mean Form Factor | VEH | P < 0.0001 | 0.2013 | Fig. 4B |
| FBs | Branch Junctions/mito | Dexamethasone 100μM | P < 0.0001 | 0.2464 | Fig. 4B |
| FBs | Branch Junctions/mito | Resveratrol 25μM | P < 0.0001 | 0.3253 | Fig. 4B |
| FBs | Branch Junctions/mito | Res 25μM +Dex 100μM | P < 0.0001 | 0.1836 | Fig. 4B |
| FBs | Branch Junctions/mito | VEH | P = 0.0005 | 0.06769 | Fig. 4B |
| FBs | MMP | Dexamethasone 100μM | P = 0.0019 | 0.04961 | Fig. 5C |
| FBs | MMP | VEH | P < 0.0001 | 0.2459 | Fig. 5C |
| FBs | ROS | Dexamethasone 100μM | P = 0.0002 | 0.067 | Fig. 5C |
| FBs | ROS | VEH | P < 0.0001 | 0.429 | Fig. 5C |
