# Supplementary figures and images for: Resveratrol and dexamethasone have cell-specific effects on the circadian clock but not on the rhythm of mitochondrial function in the fetal heart
Source: J Physiol Biochem. 2026 Feb 16;82(1):11. doi: 10.1007/s13105-026-01152-8 (PMC12909427; doi:10.1007/s13105-026-01152-8)

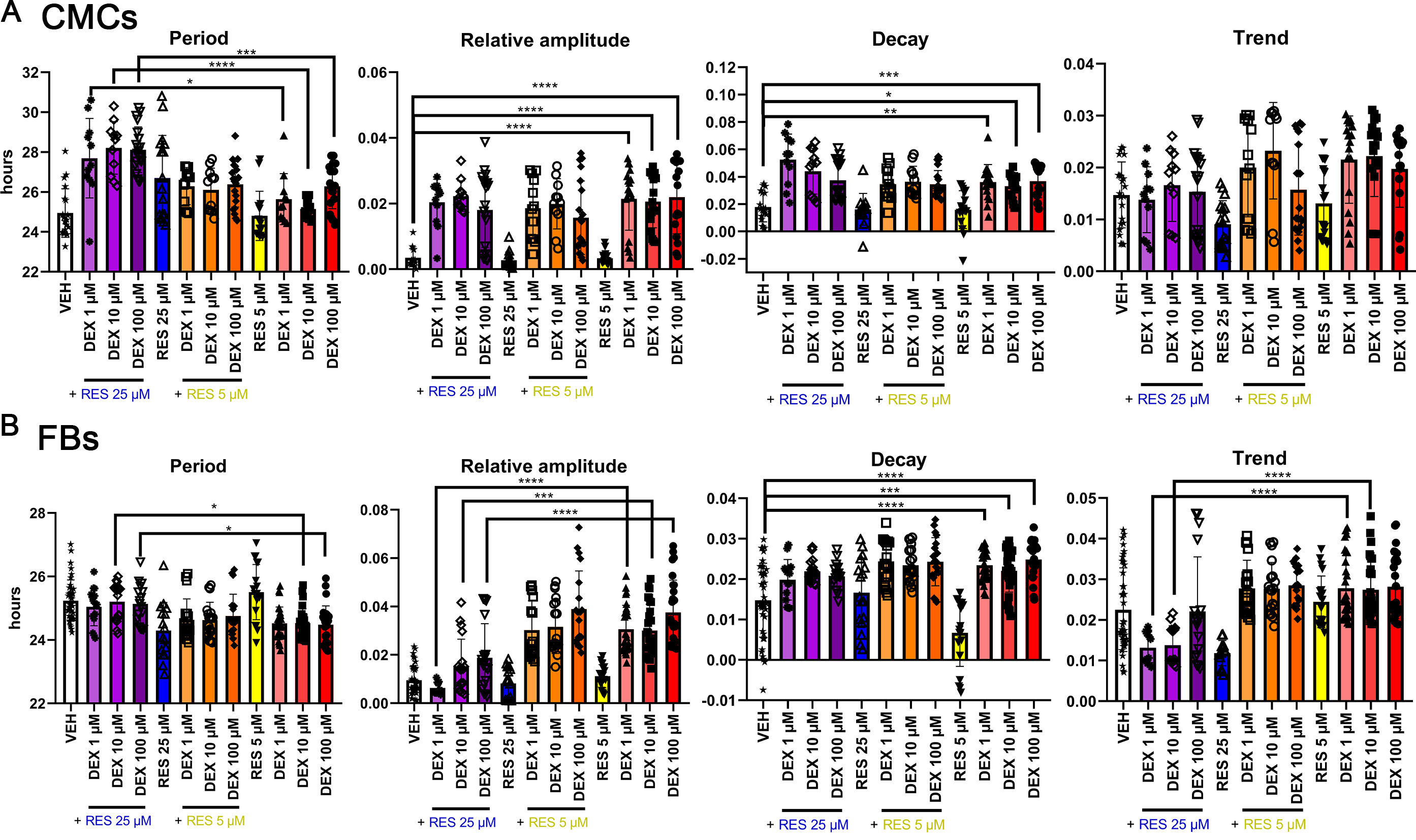

Supplement: Supplementary file 3 — Supplementary Fig. 1. Dose-dependent effect of RES on oscillatory parameters in FBs and CMCs. Isolated cells were seeded at 60,000 cells/100 μl and, after synchronization, were treated with DEX (100, 10 or 1 μM), co-treated with RES (5 or 25 μM), and VEH. The bioluminescence parameters alterations were evaluated by comparing the period, amplitude, decay, and trend of the experimental groups in CMCs (A) and FBs (B). Cells were isolated from pooled seven or more fetal hearts from each of six pregnant mice. Data were compared using 1-way ANOVA with Tukey’s multiple comparison test and presented as individual ratios and mean ± SD. Asterisks show the results of Tukey's multiple comparisons test between treatment groups. ∗P < 0.05, ∗∗P < 0.005, ∗∗∗P < 0.0005, ∗∗∗∗P < 0.0001. (PNG 721 kb) [file 13105_2026_1152_MOESM3_ESM.png]

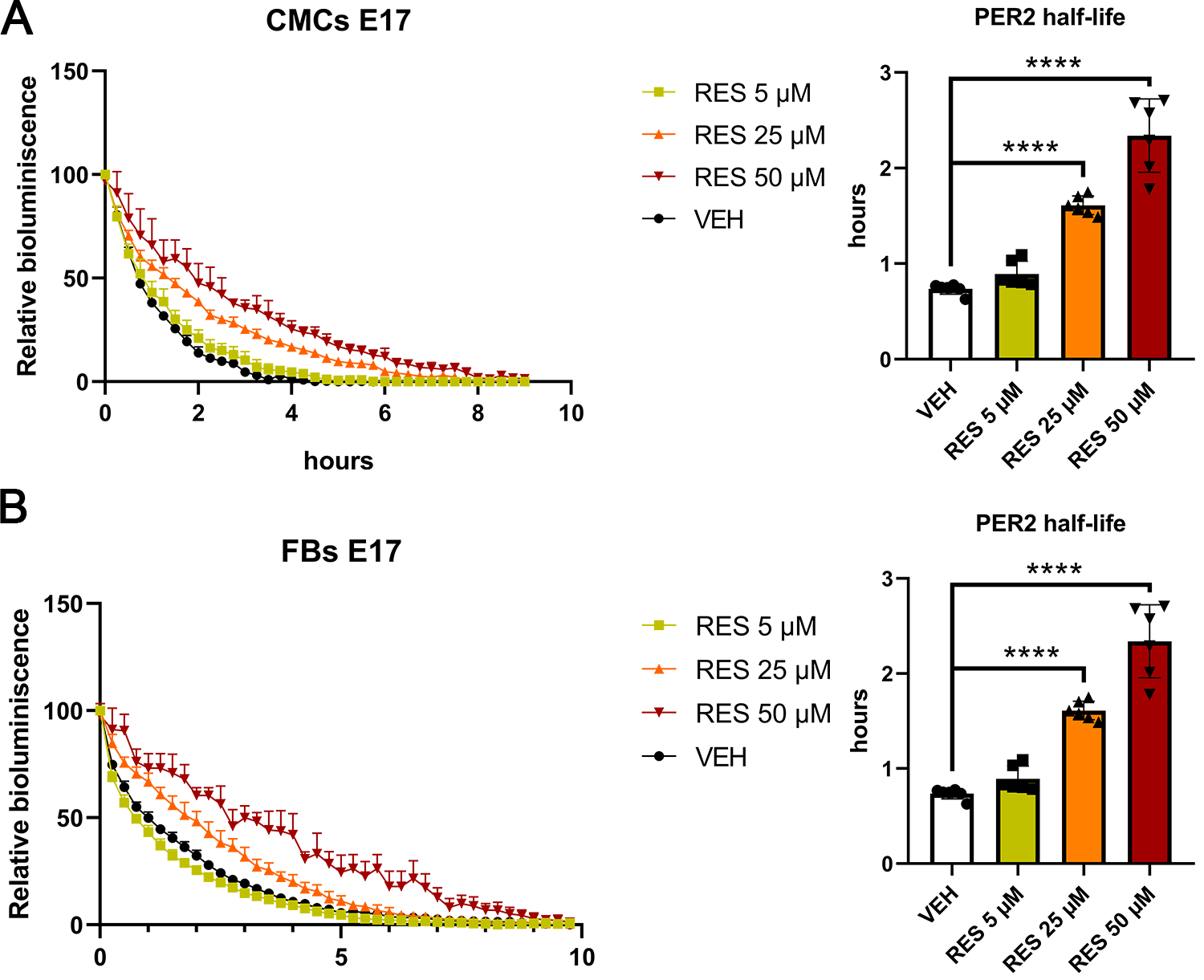

Supplement: Supplementary file 4 — Supplementary Fig. 2. RES prolongs PER2 half-life in a dose-dependent manner. Cells were isolated from at least seven fetuses from each of three pregnant mice and seeded at 60,000 cells/100 μl. CMCs (A) and FBs (B) were treated with 5, 25 and 50 μM RES or VEH. Treatment with cycloheximide (40 μg/ml) was done after reaching peak of bioluminescence with subsequent recording of PER2 degradation curve (A and B, left graphs). Half-life was analysed using 1-way ANOVA with Tukey multiple comparisons test (A and B, right graphs). ∗P < 0.05, ∗∗P < 0.005, ∗∗∗P < 0.0005, ∗∗∗∗P < 0.0001. (PNG 221 kb) [file 13105_2026_1152_MOESM4_ESM.png]

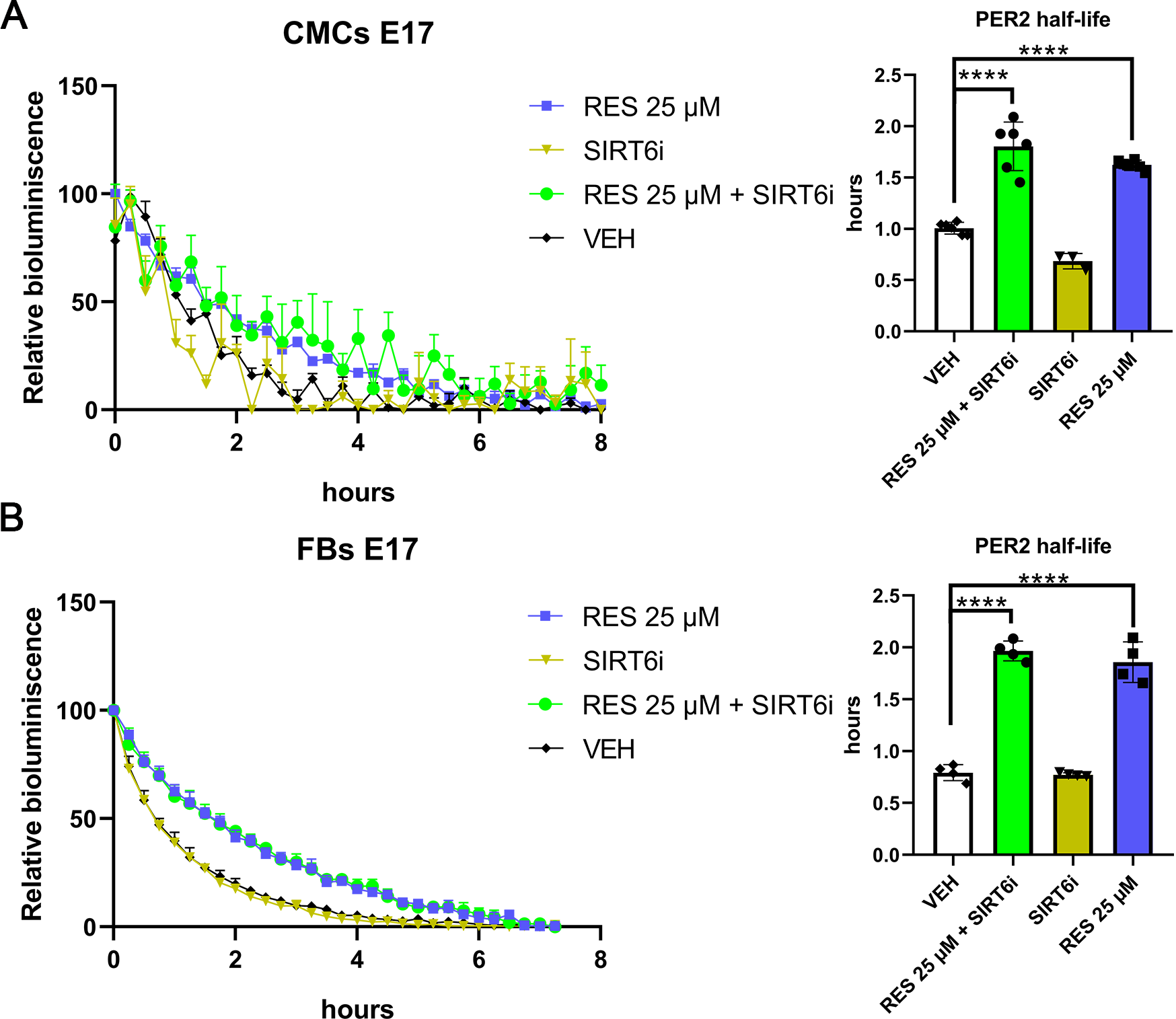

Supplement: Supplementary file 5 — Supplementary Fig. 3. SIRT6 inhibitor (SIRT6i) did not reduce RES-increased PER2 half-life. CMCs and FBs were isolated from at least seven fetuses from each of three pregnant mice. Cells were seeded at 60,000 cells/100 μl and subsequently treated with 25 μM RES, co-treated with 150 μM SIRT6i (OSS_128167) or VEH. Upon reaching a peak of the bioluminescence, cells were treated with cycloheximide (40 μg/ml) following recording of PER2 degradation curve in CMCs and FBs (A and B, left graphs). PER2 half-life was quantified by fitting exponential decay curves to the first 8 h of luminescence data post-CHX treatment. Half-life was analysed using 1-way ANOVA with Tukey multiple comparisons test (A and B, right graphs). ∗P < 0.05, ∗∗P < 0.005, ∗∗∗P < 0.0005, ∗∗∗∗P < 0.0001. (PNG 299 kb) [file 13105_2026_1152_MOESM5_ESM.png]

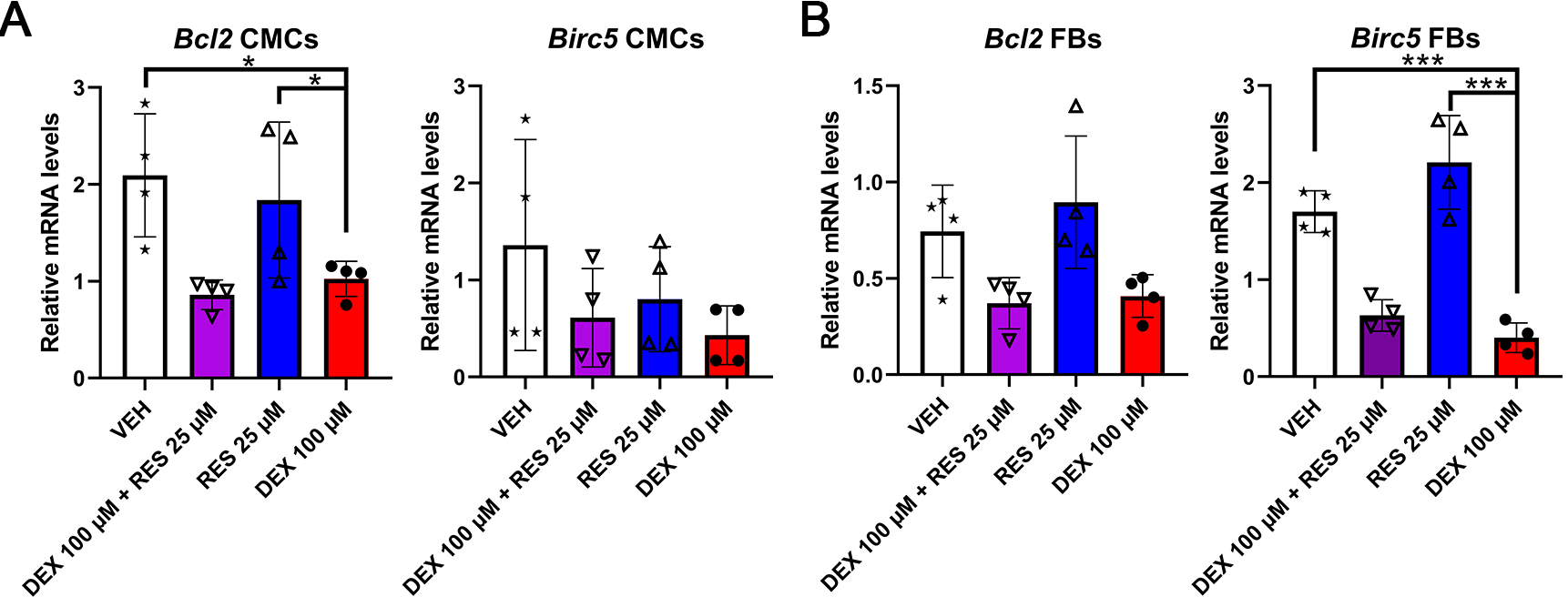

Supplement: Supplementary file 6 — Supplementary Fig. 4. The level of markers of mitochondrial function was downregulated after DEX application in CMCs and FBs. To study the effect of RES and DEX treatments on mitochondrial metabolism, RT-qPCR assay was done to detect the level of Bcl2 and Birc5 genes in CMCs (C) and FBs (D). Cells were isolated from pooled seven or more fetal hearts from each of four pregnant mice. Both CMCs and FBs were seeded at 60,000 cells/100 μl. Statistics were analyzed by 1-way ANOVA with Dunnett's multiple comparisons test to VEH. ∗P < 0.05, ∗∗P < 0.005, ∗∗∗P < 0.0005, ∗∗∗∗P < 0.0001. (PNG 134 kb) [file 13105_2026_1152_MOESM6_ESM.png]
